# Large Visual Stimuli Induce Two Distinct Gamma Oscillations in Primate Visual Cortex

**DOI:** 10.1523/JNEUROSCI.2270-17.2017

**Published:** 2018-03-14

**Authors:** Dinavahi V.P.S. Murty, Vinay Shirhatti, Poojya Ravishankar, Supratim Ray

**Affiliations:** ^1^Centre for Neuroscience, Indian Institute of Science, Bangalore, India 560012, and; ^2^Indian Institute of Science Mathematics Initiative, Department of Mathematics, Indian Institute of Science, Bangalore, India 560012

**Keywords:** area V1, EEG, gamma, LFP, oscillation, rhythm

## Abstract

Recent studies have shown the existence of two gamma rhythms in the hippocampus subserving different functions but, to date, primate studies in primary visual cortex have reported a single gamma rhythm. Here, we show that large visual stimuli induce a slow gamma (25–45 Hz) in area V1 of two awake adult female bonnet monkeys and in the EEG of 15 human subjects (7 males and 8 females), in addition to the traditionally known fast gamma (45–70 Hz). The two rhythms had different tuning characteristics for stimulus orientation, contrast, drift speed, and size. Further, fast gamma had short latency, strongly entrained spikes and was coherent over short distances, reflecting short-range processing, whereas slow gamma appeared to reflect long-range processing. Together, two gamma rhythms can potentially provide better coding or communication mechanisms and a more comprehensive biomarker for diagnosis of mental disorders.

**SIGNIFICANCE STATEMENT** Gamma rhythm has been associated with high-level cognitive functions such as attention and feature binding and has been reported to be abnormal in brain disorders such as autism and schizophrenia. Unlike previous studies that have shown a single gamma rhythm in the primate visual cortex, we found that large visual gratings induce two distinct gamma oscillations in both monkey LFP and human EEG. These rhythms, termed slow (25–45 Hz) and fast (45–70 Hz), exhibited distinct tuning preferences, latencies, and coherence profiles, potentially reflecting processing at two different ranges. Multiple gamma oscillations in visual cortex may provide a richer representation of external visual stimuli and could be used for developing brain–machine interfacing applications and screening tests for neuropsychiatric disorders.

## Introduction

Gamma rhythm (30–70 Hz) has been associated with high-level cognitive functions (Fries et al., 2001; Jensen et al., 2007; Gregoriou et al., 2009; Tallon-Baudry, 2009) and is abnormal in some mental disorders (Uhlhaas and Singer, 2012), making it a potentially valuable signal to study brain function in health and disease (Herrmann and Demiralp, 2005; Fries, 2009; Buzsáki et al., 2013). Recent studies have shown the existence of two (Colgin et al., 2009) or three (Belluscio et al., 2012) distinct gamma oscillations in the hippocampus, which are preferentially coupled to different brain areas (Colgin et al., 2009) and also to different phases of the theta rhythm (Colgin et al., 2009; Belluscio et al., 2012). In the primary visual cortex (V1), gamma has been studied extensively using visual stimuli such as bars and gratings in the context of binding through synchronization of relevant neural populations (Eckhorn et al., 1988; Gray et al., 1989; Gray and Prisco, 1997; Singer, 1999). This rhythm is highly dependent on stimulus features such as size, contrast, and spatial/temporal frequency (Henrie and Shapley, 2005; Gieselmann and Thiele, 2008; Ray and Maunsell, 2010, 2011; Jia et al., 2011, 2013; Hadjipapas et al., 2015) in the case of gratings. However, in contrast to hippocampal gamma, even though more than one gamma band can occasionally be observed in some reports (see Discussion for more details), most studies have reported a single gamma rhythm that is sustained throughout the stimulus period. However, a recent modeling study has shown that, in some regimes, a network with two inhibitory subpopulations can produce two gamma oscillations (Keeley et al., 2017). Consistent with this notion, a recent study (Veit et al., 2017) in mouse V1 has shown that large gratings engage long-range inhibitory somatostatin interneurons that produce a size-dependent gamma whose frequency is lower than the gamma associated with the parvalbumin-positive GABAergic interneurons. We therefore tested whether it is possible to generate two gamma oscillations in area V1 by presenting large visual stimuli (>10° of visual field) that could engage large neuronal populations, thereby potentially activating distinct inhibitory subpopulations in the brain (Adesnik et al., 2012; Veit et al., 2017).

Large stimuli have been used in human EEG or MEG studies (Herrmann and Demiralp, 2005; Swettenham et al., 2009; Muthukumaraswamy and Singh, 2013; Orekhova et al., 2015), but studies using microelectrodes in monkeys have typically used smaller stimuli positioned on the center of the receptive field of V1 neurons (Henrie and Shapley, 2005; Berens et al., 2008; Gieselmann and Thiele, 2008; Jia et al., 2011; Ray and Maunsell, 2011). Such “small” stimuli usually stimulate the classical receptive field and the extraclassical surround region of the V1 receptive field (typically 1–4° of visual field), whereas “large” stimuli (usually 4° and above), typically used in EEG and MEG studies, produce more widespread visual stimulation. However, tuning preferences of gamma generated by such large visual stimuli in LFPs obtained from monkeys or EEG obtained from humans or monkeys have not been well characterized. Here, we recorded monkey LFP in V1 using chronic arrays and human/monkey EEG while presenting full screen sinusoidal grating stimuli that varied in orientation, contrast, drift speed, and spatial frequency and studied the tuning preferences of induced gamma oscillations.

## Materials and Methods

### 

#### 

##### Animal recordings.

All the animal experiments were performed in compliance with the guidelines approved by the Institutional Animal Ethics Committee of the Indian Institute of Science and the Committee for the Purpose of Control and Supervision of Experiments on Animals. Two adult female bonnet monkeys (*Macaca radiata*; 3.3 and 4 kg) were used in this study. For each monkey, a titanium head post was implanted surgically under general anesthesia followed by a period of training in a visual fixation task. After the monkeys were sufficiently trained, they were operated under general anesthesia and implanted with a 10 × 10 array of microelectrodes (96 active platinum electrodes, Utah array; Blackrock Microsystems) in area V1 of the right cerebral hemisphere (∼15 mm rostral from the occipital ridge and ∼15 mm lateral from the midline). The microelectrodes were 1 mm long and the interelectrode distance was 400 μm. The reference wires were placed over the dura near the recording sites or wrapped around titanium screws on the surface of the skull near the craniotomy. The receptive fields of the neurons recorded from the microelectrodes were centered in the lower left quadrant of the visual field at an eccentricity of ∼3° to ∼4.5° in Monkey 1 and ∼1.4° to ∼1.75° in Monkey 2. As in our previous studies, only electrodes for which reliable estimates of receptive field centers were obtained and for which the impedances were between 250 and 2500 KΩ were selected for further analysis, yielding 65 electrodes for Monkey 1 and 34–36 electrodes for Monkey 2 (some electrodes showed high impedance on some sessions for Monkey 2 and were not considered for analysis for that session).

Raw signals from 96 channels were recorded using the 128-channel Cerebus Neural Signal Processor (Blackrock Microsystems). Signals were filtered between 0.3 Hz (Butterworth filter, first order, analog) and 500 Hz (Butterworth filter, fourth order, digital), sampled at 2 kHz, and digitized at 16 bit resolution to obtain the LFP signals. Raw signals were separately filtered between 250 Hz (Butterworth filter, fourth order, digital) and 7.5 kHz (Butterworth filter, third order, analog) and subjected to a threshold (amplitude threshold of ∼5 SDSs of the signal), followed by spike sorting (see below for details) to extract the multiunits.

Monkey EEG was recorded using two passive silver disc electrodes (Grass Technologies) simultaneously with LFP from scalp regions that approximately corresponded to the occipital and parieto-occipital areas referenced to an electrode placed more centrally. Acquisition system and settings were the same as that for LFP recordings. There were slight differences in electrode placements in the two monkeys due to differences in head sizes, location of the microelectrode connector, and the presence of titanium plates, mesh, and screws on the skull that were used to secure the craniotomy and the wire connecting the array and the connector.

##### Human recordings.

A total of 19 subjects (9 males and 10 females, aged between 21 – 29 years, mean age at 24.9 years) were recruited from the student community of the Indian Institute of Science for the experiments on a voluntary basis against monetary compensation. Informed consent was obtained from all the subjects for performing the experiment. All procedures were approved by the Institute Human Ethics Committee of the Indian Institute of Science. Three subjects had noisy EEG signals (flat power-spectral density curves), whereas one subject showed no appreciable increase in power from the baseline in either of the gamma rhythms at any spatial frequency studied. These four subjects (two males and two females) were not considered for further tuning experiments.

Raw EEG signals were recorded from 64 active electrodes (actiCAP) using BrainAmp DC EEG acquisition system (Brain Products). Electrode placement was according to the international 10–10 system. Raw signals were filtered online between 0.016 Hz (first-order filter) and 1000 Hz (fifth-order Butterworth filter), sampled at 2500 Hz, and digitized at 16-bit resolution (0.1 μV/bit). In a subset of subjects (four of 15), EEG for the spatial frequency tuning experiment was recorded using the 128-channel Cerebus Neural Signal Processor (Blackrock Microsystems) and a passive electrode system (BrainCap; Brain Products) using the same recording parameters as those for monkeys (64 of the available 128 channels were recorded using the international 10–10 system). Signals did not differ qualitatively across the two setups. Impedance of all electrodes was kept <5 kΩ for all subjects for all experiments except for subjects S1 and S15 for whom the impedance was kept <10 kΩ.

**Figure 1.**
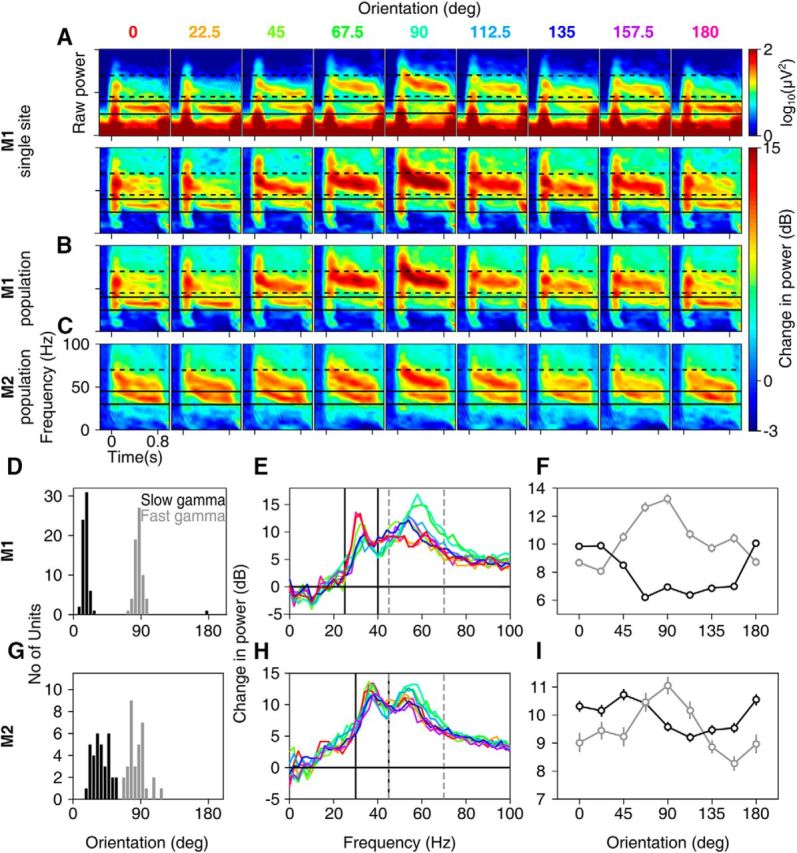
Orientation tuning of slow and fast gamma oscillations in macaque monkeys. Time–frequency difference spectra for 9 grating orientations (labeled above the plots in degrees; stimulus is presented during 0–0.8 s) for (bottom row; the corresponding raw time–frequency power spectra are shown in the top row) an example site in Monkey 1 (***A***), averaged across 65 sites in Monkey 1 (***B***) and 34 sites in Monkey 2 (***C***). Solid and broken black lines show slow and fast gamma ranges. ***D***, Histogram of orientation preference of slow and fast gamma across 65 sites in Monkey 1. ***E***, Average change in power from baseline (−0.5 to 0 s) to the stimulus period (0.25–0.75 s) across frequencies for each orientation. ***F***, Average change in power in the slow and fast gamma as a function of orientation. ***G***–***I***, Same as ***D***–***F*** but for 34 sites in Monkey 2. For all figures, error bars indicate SEM and are smaller than the size of the symbols when not visible. Figure 1-1 shows orientation tuning in monkey EEG. Figure 1-2 shows time–frequency difference spectra and change in power spectra up to 150 Hz to depict the harmonic of fast gamma in monkey LFP.

**Figure 2.**
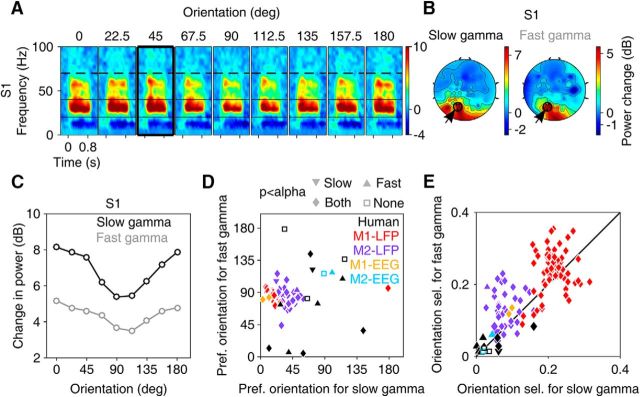
Slow and fast gamma oscillations in human EEG. ***A***, Change in time–frequency power spectrum from baseline (−0.5–0 s) for an example subject (S1). Power is averaged across three bipolar pairs in the left occipital and parietal area, shown as black dots (encircled and pointed by an arrow) in ***B***. ***B***, Scalp maps for slow and fast gamma ranges for stimulus orientation of 45° (highlighted with a black box in ***A***). Similar time–frequency difference spectra and scalp maps for the rest of the subjects is shown in Figure 2-1. ***C***, Change in power from baseline for nine orientations for S1. ***D***, ***E***, Preferred orientations (***D***) and orientation selectivity (***E***) for slow and fast gamma rhythms for 12 human subjects, monkey EEG (2 sites per monkey) and monkey LFP (65 and 34 sites). Different symbols in ***D*** and ***E*** indicate statistical significance for orientation selectivity (calculated from original data) compared against randomly permuted data (see “Statistical analysis” section in Materials and Methods for details) for slow and fast gamma (as indicated above ***D***). Significance level (α) is Bonferroni corrected (from 0.05) for number of human subjects or electrodes (for monkeys). Figure 2-2 shows results for orientation tuning after data containing microsaccades are discarded from analysis.

EEG signals were referenced to FCz during acquisition, but the data at each electrode were re-referenced offline to its neighboring electrodes using a bipolar reference scheme. We chose the bipolar referencing scheme because it yielded less noisy time–frequency spectra and a stronger gamma-band response in most subjects compared with unipolar referencing, although two gamma bands were observed in most subjects with either referencing scheme. Gamma power was most concentrated on occipital and some parietal electrodes, although there was considerable variability across subjects and even between the two hemispheres in a subject (Fig. 2-1). Similarly, although two gamma bands were visible for most subjects, there were minor variations in the center frequencies and bandwidths. Optimization of electrodes and gamma ranges (and also time period over which power was computed because there was some variability in the interval over which gamma was observed) for each subject yielded a stronger gamma response, but it also posed issues with statistical comparison because more free parameters were available to optimize gamma. We therefore used an extremely conservative approach. First, we fixed the slow and fast gamma ranges to 20–40 and 40–70 Hz, respectively, for all subjects because the two gamma peaks were mainly localized within these ranges (although sometimes the peaks were slightly off; e.g., the slow gamma for Subject S9; Fig. 2-1). Second, the time period for computation of power was set to 250–750 ms (same as for monkeys). Finally, we only considered the mean power of three bipolar combinations: P1–PO3, P3–PO3, and O1–PO3 on the left side and P2–PO4, P4–PO4, and O2–PO4 on the right side and used the side that showed more change in power for analysis. Results were similar when data were pooled across sides, although the effects were weaker.

To analyze the tuning properties of both fast and slow gamma, we used subjects in whom the power increased by at least 0.5 dB from baseline in both gamma bands, yielding 12 subjects (S1–S12).

##### Experimental setting and behavioral task.

For the behavioral task, the monkeys sat in a chair inside a Faraday cage enclosure (used for shielding from external electrical noise) with their head fixed by the head post. Human subjects sat in a separate room with their head supported by a chin rest. The Faraday cage was used only for those human subjects in whom passive electrodes were used.

The macaques and human subjects performed the same visual fixation task in which they were required to hold their gaze within 2° (for macaques) or 5° (for human subjects) of a small fixation spot (0.05° or 0.1° for monkeys and 0.1° for humans) shown at the center of a gamma-corrected LCD monitor screen (BenQ XL2411, 1280 × 720 resolution, 100 Hz refresh rate). For macaques, the monitor was placed at a distance of 50 cm from their eyes such that full screen gratings spanned a width and height of ∼56° and ∼33° of visual field. The monitor was placed 57–63 cm from the eyes of human subjects (according to their convenience; width of at least 46.8° and height of at least 27.2° of visual field for full screen gratings). The stimuli were calibrated to the viewing distance in all cases.

Every trial started with the onset of a fixation spot on which the subjects were required to hold and maintain fixation. After an initial blank period of 1000 ms, a series of two to three stimuli were shown for 800 ms each with an interstimulus interval of 700 ms. Monkeys were rewarded with a drop of juice if fixation was maintained throughout the trial.

##### Stimuli.

The stimuli were sinusoidal luminance gratings presented full screen or as circular patches of a specified size. For monkeys, for studying the spatial frequency and orientation tuning, full-screen static gratings were shown at full contrast at 1 of 5 spatial frequencies: 0.5, 1, 2, 4, and 8 cycles per degree (cpd) and 1 of 9 orientations: 0°, 22.5°, 45°, 67.5°, 90°, 112.5°, 135°, 157.5°, and 180°. Contrast tuning and size tuning (for static gratings) and temporal frequency tuning (for drifting gratings) were then studied separately for the combinations of spatial frequency and orientation that produced the highest power in the slow gamma (2 cpd and 0° for Monkey 1; 2 cpd and 45° for Monkey 2) and the fast gamma (2 cpd and 90° for Monkeys 1 and 2) frequency bands. The following 9 contrasts (presented full screen) were included in the contrast tuning study: 0%, 12.5%, 25%, 37.5%, 50%, 62.5%, 75%, 87.5%, and 100%. For the temporal frequency tuning study, full-screen, 100% contrast gratings were drifted at the following frequencies: 0 (static grating for comparison), 0.5, 1, 2, 4, 8, and 16 cycles per second (cps). For the size-tuning study, the following diameters were used for both monkeys: 1°, 2°, 4°, 8°, 16°, 32°, and full screen centered on the receptive field of one site near the center of the grid.

For human subjects, the experiments were performed in two different sessions. In the first session, spatial frequency tuning was tested using full-screen static gratings at full contrast presented at 5 spatial frequencies (0.5, 1, 2, 4, and 8 cpd) and 4 orientations (0°, 45°, 90°, and 135°). Time–frequency plots were calculated for each combination of spatial frequency and orientation from the data pooled across five occipital and parieto-occipital electrodes that showed maximum change in power from baseline between 20 and 50 Hz and the spatial frequency for which gamma was prominent and sustained throughout the stimulus period was selected for the remaining tuning experiments. Next, an orientation-tuning experiment was performed with nine orientations (same as monkey experiments), of which one orientation was selected for further tuning experiments based on similar criteria as the spatial frequency tuning experiment. The contrast, temporal frequency, and size tuning experiments were conducted in the second session with the same values as the monkey experiments except that the stimuli were centered at the fixation point for the size-tuning experiment.

##### Artifact rejection.

We discarded data for which the waveforms within a defined time period of −700 to 800 ms of stimulus onset exceeded a threshold in all the electrodes (typically due to a movement or electrical artifact). We also calculated mean amplitude of the waveforms both within and across repeats in the defined period and discarded those that exceeded a threshold of six times the SD from the mean. For monkey LFP, this led to an average rejection of <1.5% of data, yielding 30.0 ± 3.9 and 30.2 ± 0.3 (minimum of 16) stimulus repeats for each condition for the two monkeys. For monkey EEG, a larger fraction of trials (14.3% and 44.6% for the two monkeys) were discarded (because of jaw-movement-related artifacts, etc.), yielding 27.5 ± 2.0 and 17.1 ± 7.1 stimulus repeats (minimum of 11) for the two monkeys.

For human data, bad stimulus repeats were first selected for each unipolar electrode in a similar way as described above for monkeys. In addition, for the purpose of bipolar referencing, for each bipolar electrode, union of bad repeats for the two constituting unipolar electrodes was considered bad for that bipolar electrode and rejected from analysis. Although this led to unequal repeats for the bipolar electrodes for any subject, there were sizeable (at least 15) repeats per stimulus per electrode for every subject during analysis, with a maximum rejection rate of ∼45% for any electrode. The exceptions to this were S5 (experiment: orientation, electrode: F5–FC5, 6 repeats, 81% rejection), S5 (TF, AF3–F3, 9 repeats, 72% rejection), S5 (SF, C1–Cz, 13 repeats, 61% rejection), S2 (orientation, F7–FT7, 8 repeats, 75% rejection), and S8 (SF, TP7–P7, 13 repeats, 48% rejection). Note that these electrodes were not in the occipital region; they were used only in the generation of the scalp maps. Number of analyzable repeats for occipital electrodes that were used for the computation of gamma power were high for each subject, with 31.2 ± 2.5 stimulus repeats and a rejection rate of 0.8 ± 1% (mean ± SD) across all subjects.

##### Data analysis.

All the data were analyzed using custom codes written in MATLAB (The MathWorks, RRID:SCR_001622). Power spectral densities (PSDs) and the time–frequency power spectra were computed using the multitaper method with a single taper using the Chronux toolbox (Mitra and Bokil, 2008) (http://chronux.org/, RRID:SCR_005547). Baseline period was chosen between −500 to 0 ms of stimulus onset, whereas stimulus period was chosen between 250 and 750 ms to avoid stimulus-onset related transients, yielding a frequency resolution of 2 Hz for the PSDs. For monkeys, for most cases, slow gamma was in the range 25–40 Hz and 25–45 Hz for Monkey 1 and Monkey 2, respectively, whereas fast gamma was between 45 and 70 Hz for both monkeys. However, because there was some variation in peak frequency with stimulus manipulation, minor changes were occasionally made in the limits for the two gamma bands to capture the corresponding gamma peaks satisfactorily across conditions. Across all experiments for both monkeys, the slow gamma band was in the range between 15 and 45 Hz, whereas the fast gamma was in the range between 35 and 88 Hz.

Power in the gamma bands was calculated by averaging the power values obtained from the PSDs in the corresponding frequency ranges, excluding 50 Hz (line noise). Change in power for each stimulus condition was calculated as follows: ΔPower_i_ = 10(log_10_ ST_i_ − BL_ave_), where ST_i_ is the power summed across the frequency range of interest for each of the gamma rhythms for stimulus condition *i* and BL_ave_ is the baseline power averaged across conditions (BL_ave_ = average(log_10_ BL_i_)) for that rhythm.

Time–frequency power spectra were calculated using a moving window of size 250 ms and step size of 25 ms, giving a frequency resolution of 4 Hz. The scalp maps shown in Figure 2*B* and Fig. 2-1 were generated using the topoplot.m function of EEGLAB toolbox (Delorme and Makeig, 2004, RRID:SCR_007292).

Preferred orientation and orientation selectivity for each subject and each electrode for the monkeys were calculated by the following formulae:

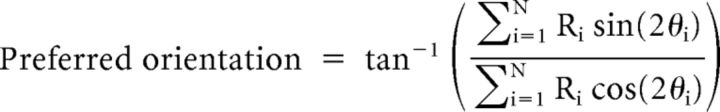


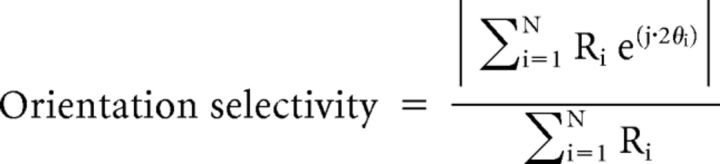
 Where θ_i_ and *R*_i_ are the orientations and power in the frequency band of interest, for each of the stimulus *i* in the orientation experiment (*N* = 8; the response at 180° was ignored because it is the same as 0° with a shifted phase). Circular variance was computed across the preferred orientation of sites using the command circ_var in CircStat (Berens, 2009).

Coherency spectrum between two signals *x* and *y* is defined as follows:

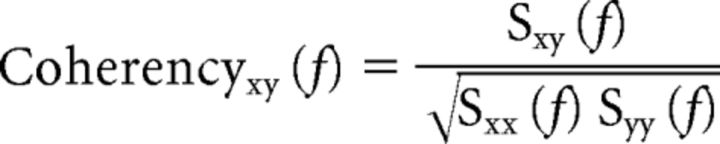
 Where *S*_xy_(*f*) denotes the cross spectrum and *S*_xx_(*f*) and *S*_yy_(*f*) denote the autospectra of each signal. These were computed using multitaper method available in Chronux toolbox using a single taper for LFP–LFP coherence. For spike–LFP coherence, five tapers were used for better visualization. Electrodes chosen for LFP–LFP coherence and the stimulus analysis period were the same as in Figure 1.

For computing spike–field coherence, units were first sorted using Spikesort (Kelly et al., 2007) (http://www.smithlab.net/spikesort.html). We chose electrodes that had at least 30 spikes in the analysis window (summed across trials) and a signal-to-noise ratio >2.5, yielding 17 and 47 sites for the two monkeys. Note that, in Monkey 2, some sites that yielded usable spikes did not show reliable LFP responses (as described above); out of the 34 sites with reliable LFP (as used in Fig. 1), 28 sites had usable spikes. Restricting the spike–field coherence analysis to only these 28 sites did not change the results. Coherency was calculated on the data obtained by pooling across all the nine orientations (as shown in Fig. 1) so that both gamma bands were well represented. However, restricting the analysis to orientations that favored slow gamma yielded similar results.

LFP–EEG coherence was computed between all the electrodes chosen for LFP analysis (65 and 34 sites for the two monkeys; Fig. 1) and each of the two EEG electrodes. For spike-EEG coherence, spike units as described above (17 and 47 units for the two monkeys) were used. For these analyses, stimulus repeats that were deemed good for both LFP and EEG (256 and 150 repeats for the two monkeys) were used.

LFP–LFP orientation tuning correlation (see Fig. 8*A*) was calculated as the Spearman's rank correlation coefficient between mean LFP responses (raw power at frequencies 0–150 Hz in steps of 2 Hz, during the stimulus period) for 8 different orientations (as used for calculation of preferred orientation) for any two sites. For these calculations, 7 tapers were used for LFP spectrum estimation for better visualization. The pairs of sites (total *N* = 2080 for Monkey 1, 561 for Monkey 2) were divided into 7 groups based on their interelectrode distances (distance ranges in mm: [0.4 0.8), [0.8 1.2), [1.2 1.6), [1.6 2.0), [2.0 2.4), [2.4 2.8) and [2.8 5.1); *N* = 197, 321, 330, 315, 364, 219, and 334 pairs for Monkey 1; *N* = 82, 130, 123, 98, 80, 36, and 12 for Monkey 2). The results were qualitatively similar when the Pearson's correlation coefficient was used as the correlation metric instead. Preferred orientation and LFP orientation selectivity at each frequency or for the gamma bands or for the spiking responses (Fig. 8*B*,*C*,*E*,*F*) were calculated as in Figure 1. Preferred orientations are depicted only for the selected sites with reliable LFP (or spiking) responses (as described earlier). For Figure 8*C*, the common sites with both reliable LFP and spiking responses are represented. This is a subset of sites shown in Figure 1*D*.

For the temporal evolution plots shown in Figure 6, the time–frequency power spectra were also computed using matching pursuit technique, which provided very good temporal resolution (Mallat and Zhang, 1993; Chandran et al., 2016), results were similar to those seen with multitaper analysis (data not shown).

##### Statistical analysis.

Parametric statistical tests (ANOVA or *t* test, wherever appropriate) were done with an assumption of normal distribution of data for monkeys as the sample size in each distribution was large (*N* = 65 and 34–36 for Monkeys 1 and 2, respectively). For humans, parametric tests were reported to maintain uniformity with monkey data, although statistical analysis performed using nonparametric tests on medians instead of means using Kruskal–Wallis test (not reported) yielded similar results. Significance level, α = 0.05, was adjusted using Bonferroni correction wherever required. Problem of multiple comparisons was avoided by appropriate grouping of distributions (e.g., to test whether mean gamma power varied across spatial frequencies, we grouped the power at 0.5 and 8 cpd as Group 1 and at 1, 2, and 4 cpd as Group 2 and performed ANOVA on the two groups).

To test whether the orientation selectivity reported in Figures 1 and 2 was significantly greater than chance, for each electrode in monkeys and for each human subject, we generated a null distribution of orientation selectivity values by randomly shuffling the orientations and recomputing the selectivity over 10,000 iterations. For slow gamma, all electrodes in Monkey 1, 33 out of 34 electrodes in Monkey 2, and 5 out of 12 human subjects had orientation selectivity significantly greater than chance (greater than the confidence level of 1–0.05/*N*, where *N* is the number of electrodes for each monkey and the number of human subjects), whereas for the fast gamma, the corresponding numbers were 65/65, 33/34, and 8/12. For Monkey EEG, selectivity was significant for both electrodes and for both gamma bands in Monkey 1, but not for Monkey 2.

##### Eye position analysis.

Eye signals were recorded using the ETL-200 Primate Eye Tracking System (ISCAN, sampled at 200 Hz) for monkeys as well as for human subjects. In five human subjects, EyeLink 1000 (SR Research, sampled at 500 Hz) was used. Stimulus presentation and monitoring of eye signals for all experiments was done by custom-made software running on MAC OS that also controlled the task flow. Mean eye positions between 0.25 and 0.75 s of stimulus onset were compared offline (using ANOVA) across different conditions in each experiment.

Although the fixation window extended to 2° from the fixation spot for monkeys (mainly to compensate for occasional slight shifts in head position during a session), the monkeys were able to maintain fixation accurately within a much smaller subregion. The SD in the eye position during the stimulus epoch across all the sessions was small for both the monkeys (0.09° and 0.11° along horizontal and vertical axes for Monkey 1; 0.16° for both axes for Monkey 2). Eye positions did not shift significantly (*p* > 0.05, ANOVA) for orientation and spatial frequency tuning experiment for both monkeys and for contrast and size tuning experiment for Monkey 1. For Monkey 2, the eye position shifts for contrast and size tuning experiments were significant, albeit very small (SD of 0.28° and 0.29° along horizontal and vertical axes for the contrast-tuning experiment; 0.31° along both axes for size tuning experiment).

For humans, although the fixation window was extended to 5°, all the subjects were able to maintain fixation with a SD of <1.2°. There was no shift of eye position across stimulus conditions for all experiments for any subject except subject S1, for whom the shift was significant, albeit small (SD of <0.3°). Because most of the tuning experiments used large stimuli, such small shifts in eye positions across conditions are unlikely to affect any of the results shown in the paper.

##### Microsaccade analysis.

Microsaccades were detected using a threshold-based method described previously (Engbert, 2006). In this procedure, eye velocities that cross a specified threshold for at least a specified duration of time are categorized as microsaccades. During a microsaccade, the peak velocity (maximum of the magnitude of velocity during the microsaccade) and peak amplitude (maximum separation between any two points during the microsaccade) are highly correlated, which is due to the ballistic nature of the microsaccade. This property is used to find appropriate velocity and duration thresholds. Specifically, these thresholds are set to maximize the correlation between peak velocity and amplitude (also called a “main sequence”, see Engbert, 2006, for details). In our data, we set the velocity threshold between 3 and 6 times the SD of eye velocities and minimum microsaccade duration between 10 and 15 ms to maximize the correlation of the main sequence for each human/monkey subject while maintaining the minimum microsaccade velocity at 10°/s and the microsaccade rate between 0.5/s and 3.0/s.

The above algorithm was applied for the analysis period of −0.5 to 0.75 s of stimulus onset for the orientation and size experiments. After removing the microsaccade-containing trials, the average number of trials available for analysis across all conditions for the orientation experiment was 17.1 and 15.8 (for LFP and EEG, respectively) for Monkey 1, 8.1 and 5.8 for Monkey 2, and 14.1 ± 1.3 for 11 humans [subject S8 had too few trials (<3) and was discarded]. For size experiment, the number of analyzable trials was 16.1 (LFP) for Monkey 1, 8.1 for Monkey 2, and 14.2 ± 0.9 for 11 humans.

## Results

We recorded monkey LFP in area V1 using chronic arrays (96 microelectrodes, Utah array, Blackrock Microsystems) from two monkeys and human EEG (64 active electrodes, BrainAmp DC, Brain Products) from 15 healthy young adults while presenting full-screen sinusoidal grating stimuli. Each stimulus was preceded by a baseline period of 700 ms and lasted for 800 ms after the onset. The monkeys and human subjects maintained fixation on a small circle at the center of the screen throughout the trial.

### Full-screen gratings induce two gamma oscillations in visual cortex with distinct tuning characteristics to orientation

We found that large (full screen) stimuli indeed generated two gamma oscillations in monkey LFP that were, surprisingly, tuned to different orientations. Figure 1*A* shows the raw (upper row) and change (lower row) in time–frequency power spectra of the LFP for nine orientations recorded from an example site from Monkey 1. Although the gamma between 45 and 70 Hz (termed “fast” gamma here) was strongest at a stimulus orientation of 90°, we observed another gamma rhythm between 25 and 40 Hz (“slow gamma”), which was strongest at 0°. Consistent with previous studies (Berens et al., 2008; Jia et al., 2011), we observed that gamma was tuned to a similar orientation across sites (Fig. 1*D*, circular variance for slow and fast gamma was 0.01 and 0.02 for Monkey 1 and 0.07 and 0.10 for Monkey 2, respectively), distinct from the tuning preference of multiunit activity (MUA) (e.g., the unit in the example site shown in Fig. 1*A* fired most strongly at 112.5°; data not shown; see Fig. 8*B*,*C* for comparison of MUA versus gamma orientation tuning across sites), which allowed us to average the change in time–frequency power spectra (Fig. 1*B*), change in power spectra (Fig. 1*E*), and overall change in power in the slow and fast gamma bands (Fig. 1*F*) across sites. Across sites, average change in power relative to spontaneous activity was maximum at ∼19° for slow and at ∼90° for fast gamma bands (Fig. 1*B*,*D–F*). Similar results were obtained for Monkey 2 (Fig. 1*C*,*G–I*), with a preferred orientation of ∼36° and ∼83° for slow and fast gamma, respectively. We also observed a small increase in the fast gamma peak frequency for more preferred orientations (Fig. 1*A*,*B*,*E*). For example, peak gamma frequencies at 90° (preferred) orientation were 58 ± 0.00 Hz (mean ± SEM) for Monkey 1 and 55.65 ± 0.21 Hz for Monkey 2, which shifted down to 51.27 ± 0.36 Hz and 52.29 ± 0.48 Hz at 45° orientation.

10.1523/JNEUROSCI.2270-17.2017.f1-1 Figure 1-1 **Orientation tuning in monkey EEG.** Same as the plots shown in Figure 1, for simultaneously collected EEG data from two electrodes in each monkey. Spectral analyses were done using 3 tapers for better visualization. Download Figure 1-1, TIF file

10.1523/JNEUROSCI.2270-17.2017.f1-2 Figure 1-2 **Harmonic of fast gamma in monkey LFP.** (A) Time-frequency difference spectra for 9 grating orientations averaged across 65 sites in Monkey 1 and 34 sites in Monkey 2 (format is same as for Figure 1B and 1C, shown up to 150 Hz). Broken black lines show range between 90-140 Hz in which the harmonic occurs, mainly for orientations around 90º. (B-C) Similar to Figure 1E and 1H, shown up to 150 Hz and using 3 tapers instead of 1 for better visualization of the harmonic. The results in the main text are shown only up to 100 Hz for better visualization of slow versus fast gamma. Download Figure 1-2, TIF file

To compare gamma oscillations recorded in LFP and EEG, we placed two electrodes near the occipital area close to the midline (electrodes could not be placed directly above the craniotomy site because the bone was secured by a titanium strap) to simultaneously record EEG from the two monkeys. For the first monkey, we observed two gamma rhythms that showed similar tuning as the LFP (Fig. 1-1). For the second monkey, fast gamma was much weaker, whereas the slow gamma was robust and strongest at ∼90°. Orientation selectivity was lower in EEG than LFP for both monkeys (see Fig. 2*E*), potentially because EEG recordings sampled a much larger cortical area.

10.1523/JNEUROSCI.2270-17.2017.f2-1 Figure 2-1 **Orientation tuning for individual human subjects.** Time-Frequency change in power plots for subjects S2 – S15 for 9 orientations of the grating stimulus. The scalp maps for the slow and fast gamma are shown next to the TF plots for each subject, computed for the orientation highlighted by a thick black box. Only subjects for which gamma power in both bands increased by at least 0.5 dB were considered for further analysis (enclosed in a rectangular box). Download Figure 2-1, TIF file

10.1523/JNEUROSCI.2270-17.2017.f2-2 Figure 2-2 **Analysis of eye-position and microsaccades for orientation-tuning experiment.** (A) Mean eye-position for monkey 1 (left plots), monkey 2 (centre) and humans (right) along the horizontal (top row) and vertical (bottom row) directions between -0.5 – 0.75 s of stimulus onset for all 9 orientations, at spatial frequency of 2 cpd for monkeys. For humans, average across 12 subjects is presented. (B) Main-sequence plots for the two monkeys and humans are presented in the top row, and histograms showing microsaccade-rate during the interval between -0.5 – 0.75 s of stimulus onset are presented in the bottom row. Data for monkeys is pooled across all spatial frequencies for maximizing the microsaccades being detected; similar results are obtained if analysis is restricted to 2 cpd. Microsaccade-rate (mean for each monkey and mean ± SEM across 12 humans), number of microsaccades (n) and correlation-coefficient (r) is indicated in the main-sequence plots. (C-D) Orientation-tuning curves for Monkeys 1 (C) and 2 (D) respectively, estimated after removing trials containing microsaccades (at least one microsaccade during the analysis period of -0.5 – 0.75 s). (E) Orientation selectivity for slow and fast gamma for the two monkeys and 11 humans calculated after removing microsaccade-containing trials. Same format as in Figure 2E. Download Figure 2-2, TIF file

Two peaks in the gamma range can be seen in many previous studies in V1 (e.g., Fig. 2*B* of Gieselmann and Thiele, 2008; Fig. 2*A* and 3*A* of Berens et al., 2008; Fig. 2*B* and 3*A* of Jia et al., 2011; and Figs. 1*H*,*I* and 2 of Ray and Maunsell, 2011). However, in these cases, the second peak is exactly at twice the frequency of the first and therefore is likely to be just a harmonic. In our data, a harmonic of the fast gamma rhythm was observed between 90 and 140 Hz in the two monkeys (Fig. 1-2, which shows Fig. 1*B*,*C*,*E*,*F* up to 150 Hz), and the power in this band was co-tuned to the fundamental (preferred orientation was ∼89° and ∼77° for the two monkeys). Conversely, fast gamma was not co-tuned with the slow gamma and did not appear at twice its frequency and therefore could not be a harmonic of the slow gamma.

Under identical stimulus conditions, we found that two gamma rhythms were observed in human EEG recordings as well. Figure 2*A* shows time–frequency difference spectra of an example subject (Subject 1) recorded from the occipital area (three black dots in Fig. 2*B*, encircled in black and indicated by an arrow). Both gamma rhythms were tuned for orientation, with a preferred orientation of ∼12° for both (Fig. 2*C*). Further, gamma was lateralized, with a larger increase on the left side for this subject (Fig. 2*B*). Despite using an extremely conservative approach to characterize gamma in the EEG (see Materials and Methods), the majority of the subjects for which orientation tuning was computed (12 of 15; Fig. 2-1) showed two rhythms that were tuned to different orientations across subjects (Fig. 2*D*). However, the mean orientation selectivity for both slow and fast gamma in human EEG was much lower than monkey LFP (mean ± SEM for slow gamma: human: 0.04 ± 0.01, Monkey 1: 0.21 ± 0.005, Monkey 2: 0.08 ± 0.005; fast gamma: human: 0.03 ± 0.006, Monkey 1: 0.24 ± 0.007, Monkey 2: 0.14 ± 0.008). Further tests based on randomization confirmed that the orientation selectivity was significantly greater than chance for almost all the LFP sites and ∼50% of the EEG subjects (5 and 8 subjects f 12, showed significant selectivity for slow and fast gamma, respectively; see Materials and Methods for details). The orientation preference of the LFP slow and fast gamma remained consistent across sessions even when separated by many days (data not shown).

A single broad gamma between 20 and 70 Hz could trivially appear as two if a broad notch filter is used to remove the line noise at 50 Hz. However, we did not use any such filter online or offline because the line noise artifact was minimal in the recordings. Further, difference in tuning preferences trivially rule out the possibility that the slow gamma was a signal processing or recording artifact because, in that case, it would be co-tuned with fast gamma. Finally, to rule out possible influences of eye movements, we tested whether eye positions or microsaccade rates varied across orientation, but did not find any systematic differences (Fig. 2-2, see Materials and Methods for more details). To discount the effect of microsaccade-related transients on gamma power, we also reanalyzed the data after removing trials containing microsaccades during the analysis period and found that orientation tuning curves remained similar for both gamma rhythms (Fig. 2-2).

### Effect of grating contrast and spatial frequency

**Figure 3.**
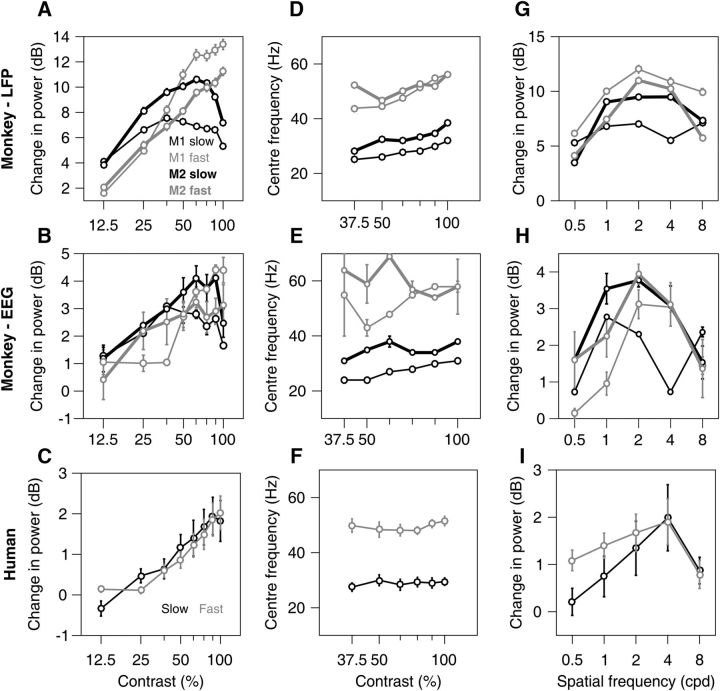
Tuning for contrast and spatial frequency. ***A***, Mean change in power for two gamma bands as a function of stimulus contrast for 65 and 36 sites for the two monkeys (top row) calculated at stimulus orientations that induced largest power change in fast gamma (90° for both monkeys) and slow gamma (0° and 45°). ***B***, Same as ***A*** but for two EEG electrodes for each of the two monkeys. ***C***, Mean change in power for 12 human subjects computed for a stimulus orientation that induced robust gamma in both bands (shown in a thick black box in Fig. 2*A* and Figure 2-1. ***D***–***F***, Mean peak gamma frequency in slow and fast bands. Same format as in ***A***–***C***. ***G***–***I***, Spatial frequency tuning for 65 and 34 sites in the two monkeys (***G***), two EEG electrodes each for the two monkeys (***H***), and 12 human subjects (***I***). Figure 3-1 shows change in power spectra for contrast and spatial frequency tuning experiments.

Next, we computed the tuning preferences of the two gamma oscillations for contrast and spatial frequency. In LFP, power of fast gamma increased with contrast (Fig. 3*A*, top), consistent with previous studies (Henrie and Shapley, 2005; Ray and Maunsell, 2010; Jia et al., 2013). Slow gamma power, however, appeared to peak for the middle contrasts (Fig. 3-1*A*, for the change in power spectra). To quantify these observations, for each electrode, we performed regression analysis between the change in power (in dB) and contrast (on a logarithmic scale with base 2). For fast gamma, the mean slopes computed for contrasts between 12.5% and 100% were 4.2 ± 0.12 dB for Monkey 1 and 3.0 ± 0.11 dB for Monkey 2, both significantly greater than zero (two-sided *t* test, *t*_(64)_ = 34.90, *p* = 2.26 × 10^−43^ for *N* = 65 and *t*_(35)_ = 26.31, *p* = 1.19 × 10^−24^ for *N* = 36 for the two monkeys). For slow gamma, we performed the analysis separately between 12.5% to 50% and 50% to 100% contrast ranges. The slopes were significantly positive for the first range (1.7 ± 0.08, *t*_(64)_ = 21.96, *p* = 1.69 × 10^−31^ and 3.2 ± 0.09 dB, *t*_(35)_ = 35.51, *p* = 4.82 × 10^−29^ for the two monkeys) and significantly negative for the second (−1.5 ± 0.12, *t*_(64)_ = −13.14, *p* = 7.81 × 10^−20^ and −2.7 ± 0.08 dB, *t*_(35)_ = −34.77, *p* = 9.88 × 10^−29^ for the two monkeys). Results were comparable for monkey EEG: slow gamma increased with first few contrast levels for both the monkeys and then decreased for the last few contrasts, whereas fast gamma showed an increasing trend for all contrasts (Fig. 3*B*). Significance for the same could not be ascertained because of the availability of only two electrodes for each monkey. For human EEG, power in both gamma bands increased with contrast (Fig. 3*C*; mean ± SEM of slopes computed over 12.5% to 100% contrast range: slow gamma: 0.76 ± 0.18 dB, *t*_(11)_ = 4.20, *p* = 0.002, fast gamma: 0.67 ± 0.15 dB, *t*_(11)_ = 4.55, *p* = 0.0008, *N* = 12), consistent with an earlier EEG report (Koch et al., 2009) that showed an increase in the change in power between 35 and 70 Hz with increasing stimulus contrast.

10.1523/JNEUROSCI.2270-17.2017.f3-1 Figure 3-1 **Change in power spectra for contrast and spatial frequency tuning in monkeys and humans.** Change in power vs frequency plots for different contrasts (A) and spatial frequencies (B) for Monkey 1 (top panels), Monkey 2 (middle) and humans (bottom). The plots for monkeys have been calculated using 3 tapers (instead of 1 as in the rest of the paper) for better visualization. For monkeys, the orientation with maximum power change in slow gamma band (left panels of A and B) and fast gamma band (right panels of A and B) was chosen. For humans, the orientation chosen for each subject is same as that used to plot scalp maps in Figure 2 and Figure 2-1. Download Figure 3-1, TIF file

Gamma peak frequency, defined as the frequency within the considered band at which the change in power was maximum, also increased with contrast in the LFP (Fig. 3*D*), consistent with previous studies (Ray and Maunsell, 2010; Jia et al., 2013) (estimated only for contrasts above 37.5% because at lower contrasts the gamma center frequency sometimes moved out of the specified range; Fig. 3-1). Regression slopes of these frequency estimates versus contrast levels were significantly positive for both the rhythms in both monkeys (mean ± SEM slopes: slow gamma: 4.6 ± 0.08 Hz, *t*_(64)_ = 55.45, *p* = 8.03 × 10^−56^ and 5.8 ± 0.32 Hz, *t*_(35)_ = 18.04, *p* = 2.64 × 10^−19^; fast gamma: 9.6 ± 0.13 Hz, *t*_(64)_ = 72.24, *p* = 4.62 × 10^−63^ and 3.3 ± 0.28 Hz, *t*_(35)_ = 12.00, *p* = 5.85 × 10^−14^; *N* = 65 and 36 for the two monkeys). For monkey EEG, peak frequency increased with contrast for slow gamma in both monkeys, although the results were inconsistent for fast gamma (Fig. 3*E*). In humans, however, peak frequency did not significantly increase with contrast (Fig. 3*F*, bottom; mean ± SEM slopes: slow gamma: 0.9 ± 1.57 Hz, *t*_(11)_ = 0.57, *p* = 0.58; fast gamma: 1.2 ± 1.32 Hz, *t*_(11)_ = 0.88, *p* = 0.40 for both gamma rhythms, *N* = 12).

For spatial frequency tuning (Fig. 3*G*), power in both gamma bands was higher between 1 and 4 CPDs than 0.5 and 8 CPD in monkey LFP (Fig. 3*G*, top plots), consistent with prior studies (Jia et al., 2011) (a comparison of mean gamma power with Group 1: 1, 2 and 4 CPD and Group 2: 0.5 and 8 CPD using ANOVA yielded highly significant *p*-values for all conditions except the slow gamma in Monkey 1, for which power remained high at 8 CPD; Monkey 1: *F*_(1,323)_ = 2.93, *p* = 0.09 and *F*_(1,323)_ = 83.65, *p* = 6.81 × 10^−18^; Monkey 2: *F*_(1,168)_ = 219.95, *p* = 2.39 × 10^−32^ and *F*_(1,168)_ = 212.48, *p* = 1.23 × 10^−31^ for slow and fast gamma, respectively). Similar trends were observed for monkey EEG, although the significance could not be determined because only two electrodes were available per monkey (Fig. 3*H*). Results were similar for humans as well, although the effect was weak (slow gamma: *F*_(1,58)_ = 3.38, *p* = 0.07; fast gamma: *F*_(1,58)_ = 5.39, *p* = 0.02; Fig. 3*I*).

### Effect of grating drift speed

**Figure 4.**
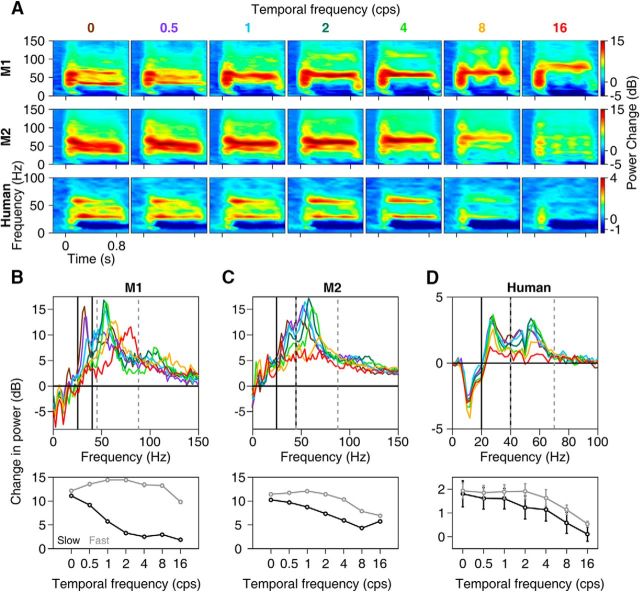
Tuning for temporal frequency. ***A***, Change in time–frequency power spectra across seven drift speeds averaged across 65 sites in Monkey 1 (top row), 36 sites in Monkey 2 (middle row), and 12 human subjects (bottom row). For monkeys, the orientation that induced the largest slow gamma (0° and 45° for the two monkeys; Figure 4-1 shows results from orientations that induced largest fast gamma) was used. ***B***–***D***, Corresponding change in power across frequency (top) and total power in slow and fast gamma bands (bottom) for Monkey 1 (***B***), Monkey 2 (***C***), and humans (***D***).

Two gamma oscillations have not been reported in V1 in earlier studies (Henrie and Shapley, 2005; Berens et al., 2008; Gieselmann and Thiele, 2008; Jia et al., 2013), although, as described in the Discussion, two peaks can occasionally be observed in some studies. Some of these studies employed drifting gratings, as opposed to stationary gratings (Henrie and Shapley, 2005; Jia et al., 2011, 2013). We thus tested the tuning of slow and fast gamma oscillations to the temporal frequency of drifting gratings. Drift speeds were varied from 0 to 16 cps (in 7 logarithmic steps) at the orientation that maximized the slow (Fig. 4) or fast gamma (Fig. 4-1). In monkey LFP, slow gamma power reduced with increasing drift speed (as depicted in tuning curves in Fig. 4*B*,*C*, bottom) and was unnoticeable for temporal frequencies of 2 cps and above for Monkey 1 and 4 cps and above for Monkey 2. In contrast, fast gamma could be observed for speeds up to at least 8 cps in both monkeys and its center frequency increased with increase in the drift speed, consistent with previous studies (Gray and Prisco, 1997; Friedman-Hill et al., 2000). In humans, the distinction between the two gammas was weaker, with both gammas showing salient change in power for drift speeds up to 4 cps (Fig. 4*A*, bottom, *D*).

10.1523/JNEUROSCI.2270-17.2017.f4-1 Figure 4-1 **Temporal frequency tuning for fast gamma in monkeys.** Same as the monkey plots shown in Figure 4, but for the orientation that induced the largest fast gamma (90º for both monkeys). Download Figure 4-1, TIF file

### Effect of grating size

**Figure 5.**
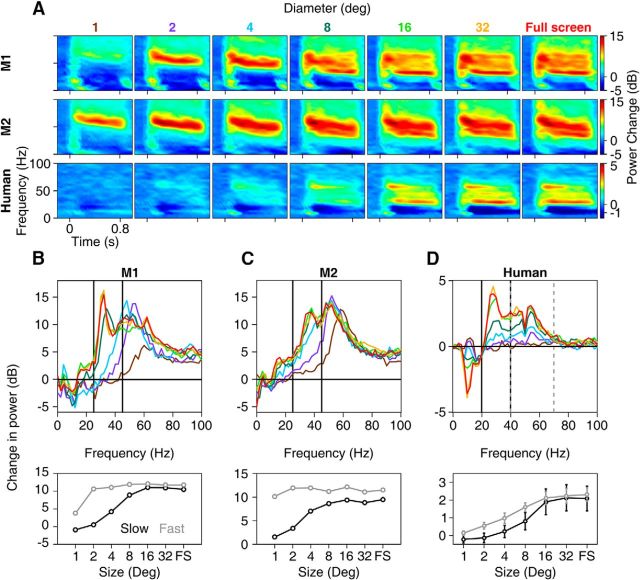
Tuning for stimulus size. Same format as in Figure 4, but for size of the grating, for 65 sites in Monkey 1, 34 sites in Monkey 2, and for 12 human subjects (bottom row). Figure 5-1 shows results from orientations that induced largest fast gamma.

Another important difference between prior studies and ours is the use of full-screen gratings instead of small gratings. We therefore tested the dependence of slow and fast gamma on stimulus size at an orientation that maximized the slow (Fig. 5) or fast gamma (Fig. 5-1). In the LFP, we found that, whereas the fast gamma appeared as soon as the stimulus covered the receptive fields of the units on the array, there was almost no or weak slow gamma for diameters <4°. Slow gamma emerged only once the stimulus diameter was 8° and above (typically, when the stimulus covered the fixation point and extended onto the other hemifield). Trends remained similar after removing trials that contained microsaccades. Results were similar in human EEG, with the fast gamma appearing before the slow (Fig. 5*A*, bottom plot). A similar increase has been noted for change in power between 30 and 70 Hz with increasing size of gratings in a recent MEG study (Perry et al., 2013).

10.1523/JNEUROSCI.2270-17.2017.f5-1 Figure 5-1 **Size tuning for fast gamma in monkeys.** Same as the monkey plots shown in Figure 5, but for the orientation that induced the largest fast gamma (90º for both monkeys). Download Figure 5-1, TIF file

**Figure 6.**
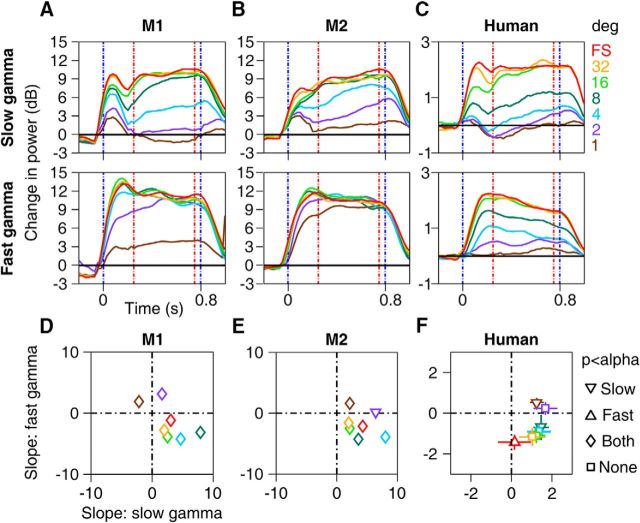
Evolution of gamma power in time. ***A***–***C***, Change in power spectra across time for slow (top row) and fast gamma (bottom row) averaged across 65 electrodes for Monkey 1 (***A***), 34 electrodes in Monkey 2 (***B***), and across 12 human subjects (***C***) computed at the preferred orientation for each gamma band. ***D***–***F***, Scatter plots showing regression slopes for slow versus fast gamma in the stimulus period (0.25–0.75 s, as indicated by red dashed lines in ***A***–***C***) for seven stimulus diameters. Error bars indicate SEM and are smaller than the size of the symbols when not visible. Different symbols indicate whether the mean slopes were significantly different from zero (*p* < 0.05, *t* test, Bonferroni corrected for the number of sizes) for slow and fast gamma (as indicated in the extreme right).

These results are consistent with the idea of the recruitment of a second, potentially larger inhibitory network by the presentation of a large stimulus, as predicted by recent models of gamma (Kang et al., 2010; Jia et al., 2013; Keeley et al., 2017). We next tested some predictions of this hypothesis. First, if the generation of slow gamma involved larger networks operating with a slower time constant (Kang et al., 2010; Jia et al., 2013) compared with fast gamma, its buildup over time should be slower than fast gamma. To test this, we plotted the gamma power as a function of time (Fig. 6), which indeed showed that power increased with time for slow gamma (Fig. 6*A–C*, top), but not for fast gamma (Fig. 6*A–C*, bottom plots; these trends are also readily observed in the time–frequency plots in Fig. 5*A*). To quantify this further, we calculated the slope of power change by performing a regression analysis between the change in power (in dB) and time (0.25–0.75 s of stimulus onset; the initial 0.25 s after stimulus onset was not considered for analysis because this included stimulus-onset-related transients that induced a broadband increase in power) separately for each electrode for the monkeys and each human subject. Mean slopes for both monkeys were significantly positive (Fig. 6*D*,*E*, *t* test, *p* < 0.05, Bonferroni corrected for multiple stimulus sizes) for grating sizes for which slow gamma was conspicuous (4° and above). We also fitted an exponential function to the power versus time traces and found that the rise time (time at which the gamma power reaches to ∼63% of saturation level) decreased with increasing grating diameters between 8° and 32° (data not shown), suggesting that buildup of slow gamma was faster with increasing stimulus size. Opposite trends were observed for fast gamma, where slopes were significantly negative during the analysis period for grating diameters of 4° and above for monkeys. Similar trends were observed in human EEG data (Fig. 6*F*), with positive mean slow gamma slopes and negative mean fast gamma slopes at all sizes above 4°, although the results failed to reach significance for certain sizes after Bonferroni correction possibly due to a smaller sample size. Results for monkey EEG were similar to those of monkey LFP for slow gamma, although the significance could not be ascertained due to insufficient sample size (two electrodes per monkey).

### Phase consistency of the two gamma rhythms across electrode distance in LFP

**Figure 7.**
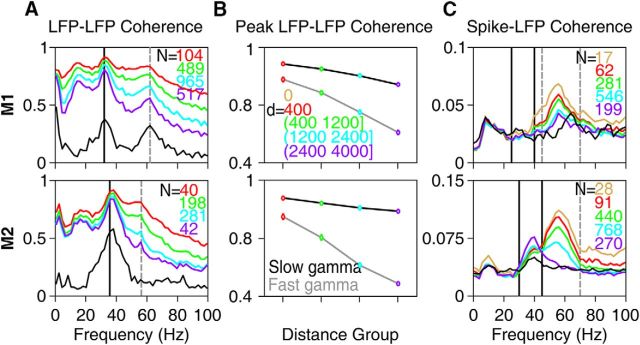
Field–field and spike–field coherence. ***A***, LFP–LFP phase coherence spectra for different interelectrode distances for Monkeys 1 (top row) and 2 (bottom row). Interelectrode distance ranges (d, in μm) are shown in ***B***. The number of pairs (*N*) for each group is indicated on the top right corner. LFP–LFP phase coherence when both are taken from the same electrode (i.e., interelectrode distance of zero) is trivially 1 at all frequencies and is therefore omitted. Mean LFP–EEG phase coherence is shown in black. ***B***, Average LFP–LFP phase coherence at the peak slow (32 and 36 Hz for the two monkeys) and fast gamma bands (62 and 56 Hz) as a function of interelectrode distance. ***C***, Mean spike–LFP coherence for spike–LFP pairs separated by different interelectrode distances. Mean spike-EEG coherence is shown in black.

If fast gamma represents more local processing than slow gamma, then its phase consistency across electrode pairs should decrease more quickly with increase in interelectrode distance compared with slow gamma. We tested this hypothesis by measuring phase coherence across sites separated into different interelectrode ranges (Fig. 7*A*). Fast gamma phase coherence indeed decreased more rapidly with interelectrode distance than slow gamma (2-way ANOVA with gamma bands and interelectrode distances as factors yielded a highly significant interaction effect: *F*_(3,4142)_ = 120.59, *p* = 7.12 × 10^−75^ and *F*_(3,1114)_ = 62.88, *p* = 1.46 × 10^−37^ for the two monkeys, Fig. 7*B*). Finally, spike–field coherence analysis (Fig. 7*C*) showed that spikes were preferentially locked to fast gamma, whereas the coupling to slow gamma was weak. However, whereas coherence decreased with interelectrode distance for the fast gamma band in a way similar to LFP–LFP coherence, slow gamma did not show any observable change. We also computed the coherence between LFP/spikes and the simultaneously recorded EEG (averaged across two occipital electrodes; black traces in Fig. 7*A*,*C*; see Materials and Methods for details). Unsurprisingly, coherence values involving EEG tended to follow qualitatively the trends observed in the corresponding LFP–LFP and spike–LFP coherences for the largest interelectrode distance range (2.4–4 mm). Similar results were obtained even when the analysis was restricted to orientations that produced stronger slower gamma compared with fast gamma (data not shown).

### Orientation tuning of the two gamma rhythms and spiking response across electrode distance

**Figure 8.**
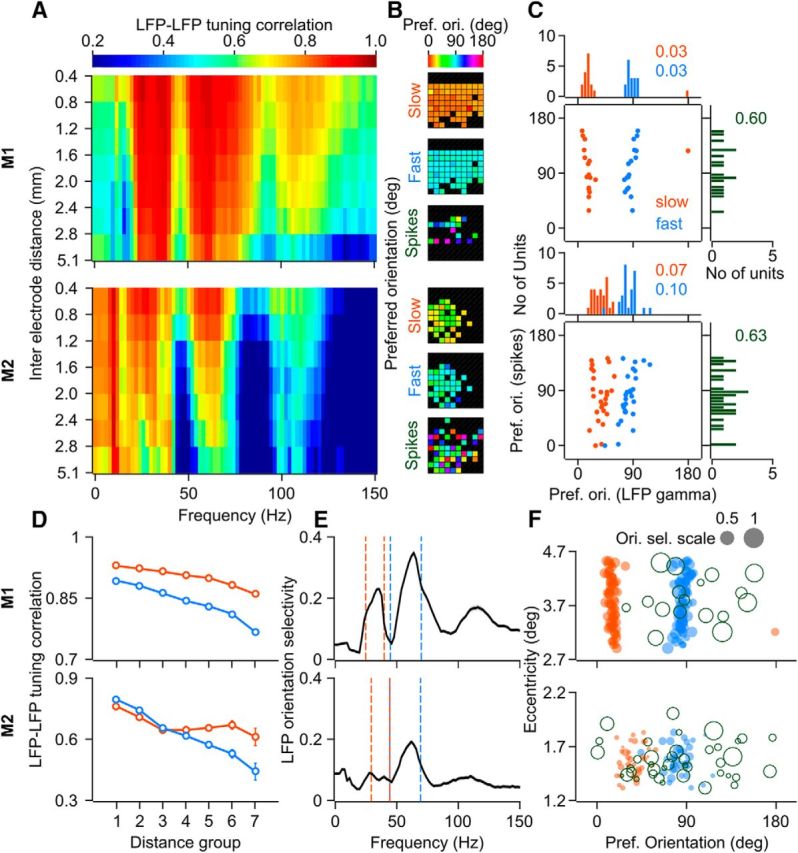
LFP and spikes orientation tuning properties across the sites. ***A***, Mean LFP–LFP orientation tuning correlation (Spearman's rank correlation coefficient) spectra for different interelectrode distances (seven groups; distance delimiters for each group are indicated on the *y*-axis, in mm) for Monkey 1 (top row) and Monkey 2 (bottom row). Total number of pairs, *N* = 2080 for Monkey 1 and *N* = 561 for Monkey 2 (groupwise numbers are provided in the Materials and Methods section). ***B***, Preferred orientation for slow gamma, fast gamma, and spiking response across the microelectrode array for the two monkeys. ***C***, Scatter of preferred orientation for slow (orange) and fast (blue) gamma versus that for the spiking response and the corresponding marginal histograms. Circular variance of the distribution of preferred orientations is mentioned in the corresponding color. *N* = 17 for Monkey 1, 28 for Monkey 2. ***D***, Mean LFP–LFP orientation tuning correlation in the slow and fast gamma bands as a function of interelectrode distance. ***E***, Mean orientation selectivity of LFP power (*N* = 65 and 34 for Monkeys 1 and 2, respectively) at different frequencies; Dashed color lines indicate the limits for slow and fast gamma bands. Gray shaded regions (barely visible) denote SEM. ***F***, Scatter of eccentricity (in degrees of visual angle) of the receptive field center of sites and the preferred orientation for the corresponding slow gamma, fast gamma, and spikes (open circles). Size of the circle denotes orientation selectivity (gray circles with the corresponding value of selectivity are shown as a reference for the scale).

Finally, we investigated whether the different scales of processing of the two gamma rhythms also reflected in their orientation tuning at sites separated by different distances. For all pairs of LFP sites, we computed the correlation of the orientation tuning curves of the LFP power and plotted the average tuning correlation at different frequencies as a function of the interelectrode distances (Fig. 8*A*, similar to Fig. 3*A* of Jia et al., 2011). The tuning correlation in the slow and fast gamma ranges (as well as at the harmonic of fast gamma at ∼110 Hz) was in general strong across both the monkeys. However, it stayed high in the slow gamma range for larger interelectrode distances than in the fast gamma range (2-way ANOVA with gamma bands and interelectrode distances as factors yielded a highly significant interaction effect: *F*_(6,4146)_ = 6.89, *p* = 2.68 × 10^−7^ and *F*_(6,1108)_ = 0.15, *p* = 1.95 × 10^−6^ for the two monkeys, respectively, Fig. 8*D*). The preferred orientation was similar across sites for both slow and fast gamma rhythms, respectively, with a low circular variance, but more distributed for the spiking responses (Fig. 8*B*,*C*) across both the monkeys. This is in agreement with previous studies showing that the orientation preference in the gamma range remains similar across sites despite the more distributed preferences of spiking responses (Berens et al., 2008; Jia et al., 2011). However, fast gamma had on average higher orientation selectivity than slow gamma (Fig. 8*E*) and was closer to the selectivity of the spiking responses, which were the most selective (mean orientation selectivity = 0.5 ± 0.06 for Monkey 1 across 17 units, 0.24 ± 0.03 for Monkey 2 across 47 units). We also investigated whether the closely clustered preferred orientations for the two gamma bands shifted stereotypically within the small range of preferred orientations across the sites according to their receptive field location, but did not find any significant and consistent trend across the two monkeys (Fig. 8*F*, linear regression of preferred orientation on eccentricity of the receptive field center of the site was not significant, *p* > 0.05 after Bonferroni correction for both gamma bands and monkeys; similar results were obtained for regression on azimuth and elevation of the receptive field centers instead of the eccentricity).

## Discussion

Our study demonstrates the presence of two simultaneous gamma oscillations, which we termed “slow” (∼20–40 Hz) and “fast” (∼40–70 Hz), in the V1 of macaques and in EEG of humans and macaques in response to large visual gratings. These rhythms were tuned to different orientations and contrasts of the gratings in LFP, with weaker effects in EEG. Slow gamma was salient only for gratings of large diameters (8° and above) that were either static or drifting at speeds <2 cps. The increase in power across time was more gradual and coherence and orientation tuning across larger interelectrode distances was more sustained for slow gamma compared with fast gamma, suggesting that fast gamma reflected local processing, whereas slow gamma reflected properties of a larger network.

### Comparison with previous studies

Whereas in the LFP, tuning for parameters such as orientation, contrast, and spatial frequency have been tested using small stimuli (Eckhorn et al., 1988; Henrie and Shapley, 2005; Berens et al., 2008; Jia et al., 2011, 2013), some studies have used relatively larger gratings (Siegel and König, 2003; Jia et al., 2011, 2013; Moca et al., 2014). Some possible reasons for not finding prominent slow gamma oscillations in these studies could be high drift speeds (Henrie and Shapley, 2005; Jia et al., 2011, 2013); insufficiently large stimuli (Berens et al., 2008; Gieselmann and Thiele, 2008); a different viewing paradigm involving eye movements (Siegel and König, 2003); anesthesia (Jia et al., 2011, 2013; Moca et al., 2014); monocular stimulation (Jia et al., 2011, 2013), which is shown to induce lesser gamma power (Eckhorn et al., 1988); or insufficient sampling of the orientation space for large stimuli (Gieselmann and Thiele, 2008). It may also be difficult to observe two gamma bands if their preferred orientations are close to each other. For example, two gamma bands are less distinguishable in time–frequency plots for Monkey 2 (Figs. 1*C*, 5*A*), although the PSD plots in Figures 1*H* and 5*C* show distinct “bumps” of slow and fast gamma.

A recent study in mouse V1 showed a “context-dependent” gamma rhythm occurring at ∼30 Hz, which was dependent on the grating size (Veit et al., 2017). Although earlier studies had implicated a role of parvalbumin-positive GABAergic neurons for the generation of traditional gamma (Bartos et al., 2007), this study showed that dendrite-targeting, long-range somatostatin interneurons are involved in modulating this slow, context-dependent gamma. A similar motif of coactivated, multiple inhibitory circuits may be involved in the generation of slow and fast gamma by large gratings that we observed in the primate V1 LFP and EEG. Further, the distinction in the tuning characteristics of slow and fast gamma suggests that diverse inhibitory subpopulations, active across different spatial scales, may be tuned differently to stimulus properties.

It is interesting that, in some EEG/MEG studies in which large gratings were used routinely, two gamma bands were observed, although the authors of these studies have not followed this observation in detail. For example, a faint slow gamma can be observed in the EEG for large moving gratings in a study by Muthukumaraswamy and Singh (2013) (see “G-4Q-M” and “A-4Q-M” conditions for EEG in their Fig. 3). Similarly, in MEG recordings, a weak slow gamma can be observed in Figure 3*B* of Hoogenboom et al. (2006), Figure 4 of Swettenham et al. (2009) (especially for the static grating stimuli), and Figure 1*B* of Orekhova et al. (2015) (especially for the moving grating stimuli for 3.6°/s case). However, slow gamma is not observed in some studies despite using full-screen gratings (Koch et al., 2009). Some differences between our study and previous work that could have led to a stronger slow gamma in our recordings include use of an appropriate stimulus orientation, Cartesian instead of annular gratings (Koch et al., 2009; Orekhova et al., 2015), bipolar referencing scheme for analysis instead of unipolar (Koch et al., 2009)/common-average (Muthukumaraswamy and Singh, 2013)/left-earlobe (Orekhova et al., 2015) reference (see Materials and Methods), and full-screen stimuli instead of smaller stimuli (Hoogenboom et al., 2006; Swettenham et al., 2009) that were not always presented in all quadrants of visual space (Swettenham et al., 2009; Muthukumaraswamy and Singh, 2013).

### Slow gamma and other rhythms in similar frequency bands

The slow gamma oscillations reported in this study fall in the same range as high beta oscillations reported in previous studies (Roopun et al., 2006). Beta oscillations have been reported mostly in somatosensory and motor systems (for review, see Engel and Fries, 2010), where they have been shown to occur prominently at rest and are attenuated by voluntary movements. These are unlike slow gamma, which is almost invisible at rest and slowly builds up with time during visual stimulation. Further, the two rhythms have been shown to be predominant in different cortical layers, with beta appearing most strongly in deep layers (Roopun et al., 2006) and gamma in superficial layers (Buffalo et al., 2011; Xing et al., 2012); because our electrodes were only 1 mm long, they are more likely to sample the superficial layers). Recent studies, however, have shown that alpha/beta rhythms could play a role in mediating feedback from higher cortical areas into visual cortex, although the frequency range in these studies was lower (peak <20 Hz) than our slow gamma range (Van Kerkoerle et al., 2014; Bastos et al., 2015; Michalareas et al., 2016). Testing whether the slow gamma reported here could also play a role in feedback would require simultaneous recordings from multiple cortical areas followed by interareal Granger causality analysis. Further, because feedback-related activity has been shown to be more prominent in infragranular layers (Van Kerkoerle et al., 2014; Bastos et al., 2015), laminar probes could be used in V1 to test whether slow gamma is strongest in infragranular layers. Although beyond the scope of this study, such experiments will elucidate a potential role of slow gamma in feedback processing.

Because gamma peak frequency reduces with increasing stimulus size, the fast gamma also occasionally enters the slow gamma range. This brings an intriguing possibility that, with increasing stimulus size, the fast gamma transitions into the slow gamma, whereas a second fast gamma originates at high frequencies. However, typically, with increasing size, the rate of falloff of gamma peak frequency reduces. For example, Gieselmann and Thiele (2008) show a shift of gamma peak frequency from ∼60 Hz to ∼40 Hz when the patch size increases from 0.1° to 1°, but a further reduction of only 5 Hz (to ∼35 Hz) when the size increases to 4° (their Fig. 3*E*). Similar results can be seen in Figure 2*A* of Jia et al. (2013). In our data, fast gamma peak frequency reduced slightly, from ∼60 Hz to ∼50 Hz, as stimulus size increased from 1° to 4° (Fig. 5*B*). This peak would have to decrease by >10 Hz going from 4° to 8° if the slow gamma that we reported is actually a “slowed down” fast gamma. Such a large jump is not consistent with the exponential falloff observed in previous studies. Nevertheless, a denser sampling of stimulus size dimension, along with a thorough characterization of tuning properties at varying sizes is needed to rule out the possibility that the slow gamma is actually the fast gamma in which the peak frequency reduces into the slow gamma range.

### Gamma rhythms in LFP versus EEG

In our data, slow gamma was typically stronger than fast gamma in EEG, but not in LFP recordings. In human EEG, fast gamma was more prominent than slow in only 3 of 15 subjects (S3, S9, and S12; Fig. 2-1). For Monkey 2, EEG recordings (Fig. 1-1) showed very weak fast gamma (prominently seen only for 90° orientation), even though it was prominent in LFP recordings. Because EEG recordings are thought to reflect activity of a large neural population, with synchronous events progressively becoming more dominant with an increase in population size (Nunez and Srinivasan, 2006), the relative prominence of slow gamma in EEG may imply a larger spatial spread compared with fast gamma.

Gamma in human EEG showed weaker selectivity for stimulus features such as orientation and spatial frequency. Consistent with a previous EEG study, we also did not observe an increase in center frequency with increasing stimulus contrast (Koch et al., 2009). Weaker selectivity could partly be due to an extremely conservative approach that we used to compute gamma power in EEG: we used the same set of electrodes, frequency ranges, and time period for analysis for all subjects (see Materials and Methods). For example, visual inspection of time–frequency power spectra of human EEG for different orientations (Fig. 2-1) shows more power at some orientations at certain time intervals and frequencies for many subjects, suggesting that orientation selectivity can be improved simply by choosing appropriate time and frequency limits (an appropriate box in the time–frequency plot) for each subject. Because power is computed at a large number of time and frequency points, it is difficult to ascertain the significance of such data-driven optimization of gamma power obtained by customizing time and frequency intervals over which gamma is computed. Because our main goal was to demonstrate the existence of two gamma bands in EEG, we have therefore used fixed intervals and electrodes for all subjects.

Gamma recorded from monkey EEG was in general better tuned than human EEG (tuning decreased from monkey LFP to monkey EEG to human EEG). However, this could simply be because the gamma ranges were more optimized for monkey EEG (same ranges as monkey LFP) than human EEG, for which a fixed frequency range was used for all subjects. Another reason could be related to the surgery performed on the monkey to implant the microelectrode array, due to which part of the skin and muscle had to be moved, potentially leading to better signal transmission to EEG electrode. EEG recordings from more monkeys, especially before the surgery and using comparable procedures for selection of gamma time and frequency ranges as humans would be needed to test whether the differences in tuning between monkey and human EEG are species specific or due to experimental differences.

Finally, we observed significant difference in the strength of gamma tuning between the two monkeys. It is unclear why the tuning is so different across monkeys, although a large variability in gamma power as well as tuning strength was observed in human EEG as well. Another reason could be because the receptive field locations were more foveal for the second monkey. It is possible that foveal locations process orientation more uniformly such that gamma is present for any orientation, leading to weaker orientation selectivity in Monkey 2. It is also unclear why fast gamma is selective for the same orientation (90°) for both monkeys or whether the preferred orientation would remain the same at different implant locations in the same monkey. Such questions are beyond the scope of this study, which would need orientation tuning data from many monkeys obtained from electrodes placed at multiple cortical locations.

### Functional significance of slow and fast gamma

Recently, Colgin et al. (2009) showed the existence of two gamma oscillations in the CA1 region of the hippocampus, with the slow gamma (25–50 Hz) preferentially coupling to the slow gamma of another hippocampal subfield called CA3, whereas fast gamma (60–140 Hz) preferentially coupled with the fast gamma recorded from layer III of the medial entorhinal cortex. Further, these oscillations preferentially occurred at different phases of the theta rhythm (Colgin et al., 2009; Belluscio et al., 2012). Two gamma rhythms have also been reported in the olfactory bulb (Kay, 2003), which occurred at different phases of the sniff cycle (Frederick et al., 2016) and were associated with different cellular networks (tufted and mitral cells) (Manabe and Mori, 2013). In these cases, two gamma rhythms that occurred at different phases of the theta/sniff cycle and coupled to different areas or cell types could serve to route information flowing into the hippocampus/olfactory bulb flexibly.

In the cortex, several studies have indicated that oscillations at lower frequencies such as alpha or beta could represent feedback from higher cortical areas, whereas gamma could represent feedforward processing (Van Kerkoerle et al., 2014; Bastos et al., 2015; Michalareas et al., 2016). Similarly, slow oscillations have been related to a larger area of information integration or communication, whereas higher frequencies have been associated with more local computations (von Stein and Sarnthein, 2000). Because slow gamma in our data was coherent over larger distances compared with fast gamma, it could potentially play a role in lateral feedback (Veit et al., 2017), although we do not provide any direct evidence.

To conclude, our findings highlight the presence of two simultaneous gamma oscillations, not only in the phylogenetically older brain structures such as the hippocampus or the olfactory bulb as reported previously, but also in the V1. These rhythms also seem to be preserved across species (Buzsáki et al., 2013) (macaques and humans tested here) and across scales (LFP and EEG). Diverse properties of these oscillations in primary sensory areas such as the visual cortex may provide a richer representation of external stimuli (orientation of gratings in this case). Further, together they may provide a more comprehensive signal for brain–machine interface applications and a more potent marker for the diagnosis of brain disorders such as autism and schizophrenia, which have been associated with abnormal gamma rhythms (Uhlhaas and Singer, 2010, 2012).
