# Supplementary figures and images for: Large Visual Stimuli Induce Two Distinct Gamma Oscillations in Primate Visual Cortex
Source: J Neurosci. 2018 Mar 14;38(11):2730–44. doi: 10.1523/JNEUROSCI.2270-17.2017 (PMC5852657; doi:10.1523/JNEUROSCI.2270-17.2017)

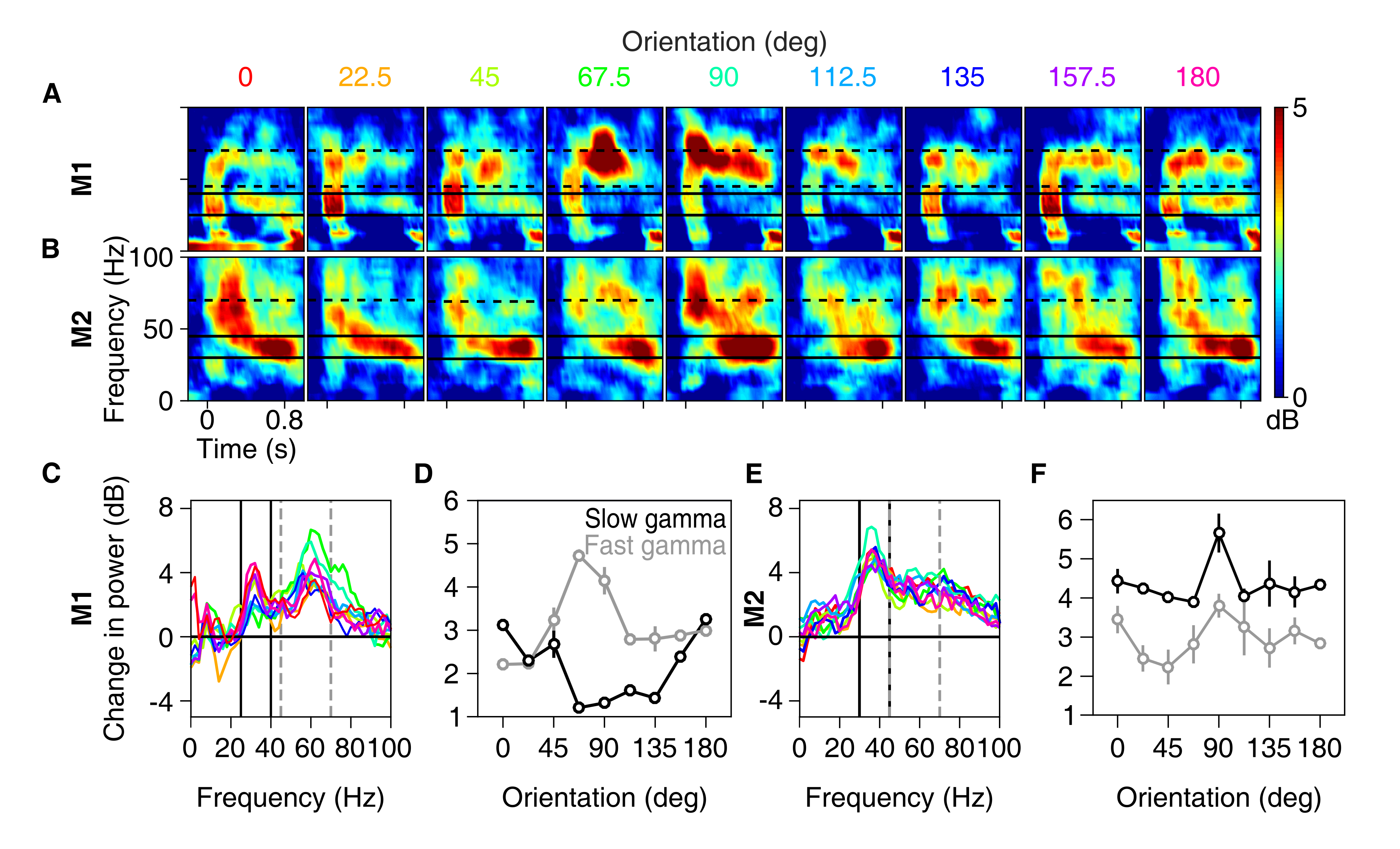

Supplement: Figure 1-1 [file zns999180605so1.tif]

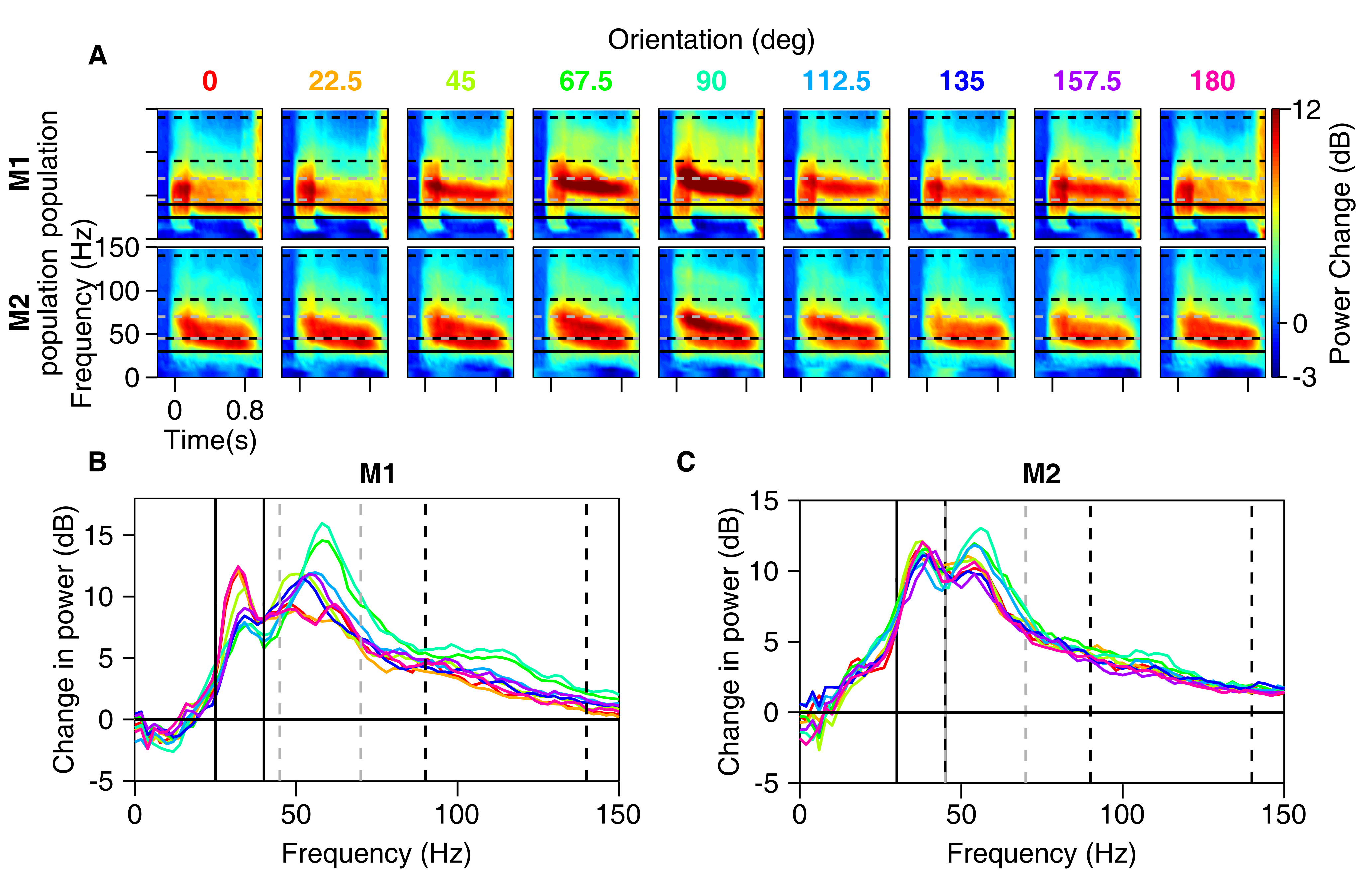

Supplement: Figure 1-2 [file zns999180605so2.tif]

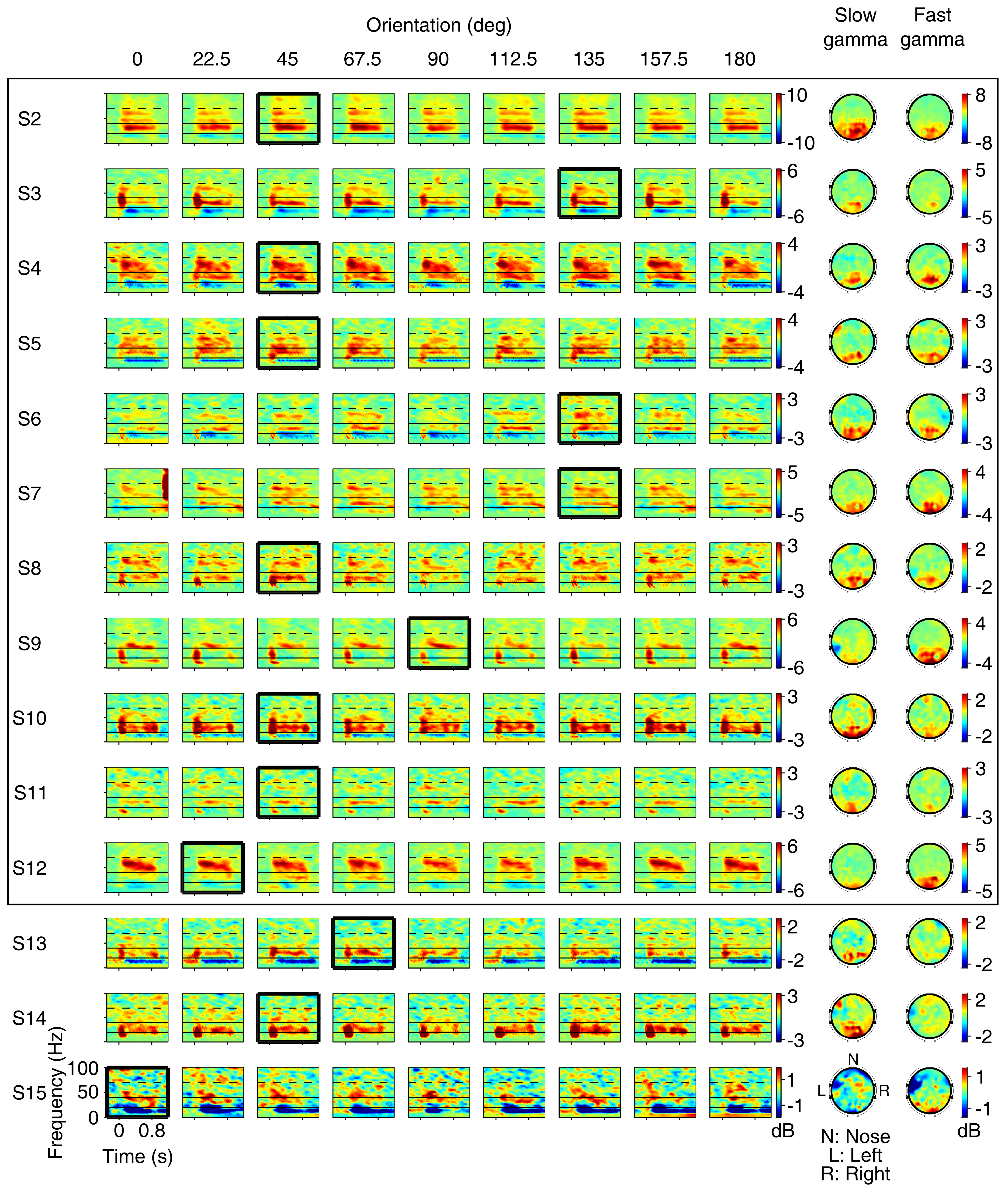

Supplement: Figure 2-1 [file zns999180605so3.tif]

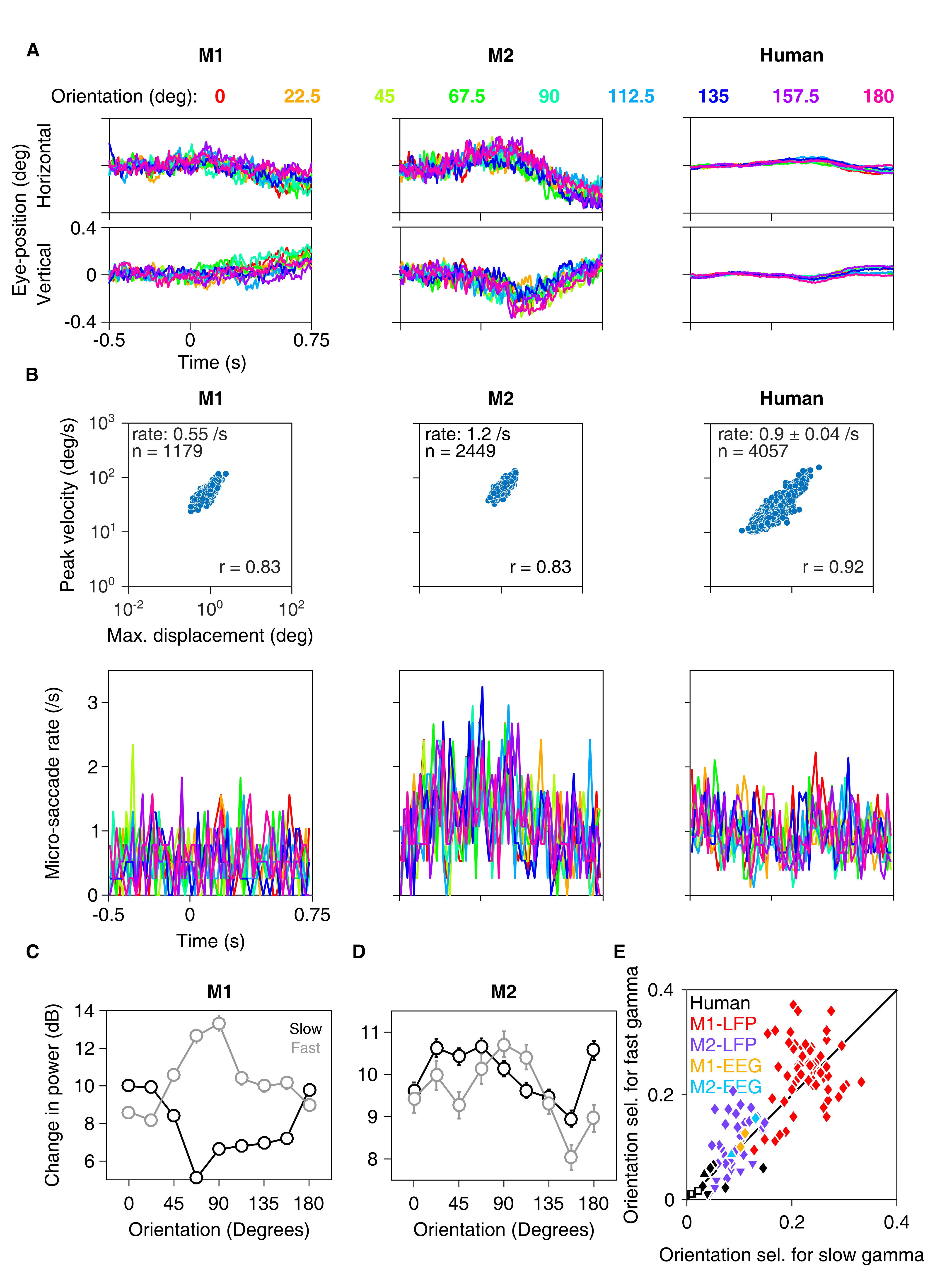

Supplement: Figure 2-2 [file zns999180605so4.tif]

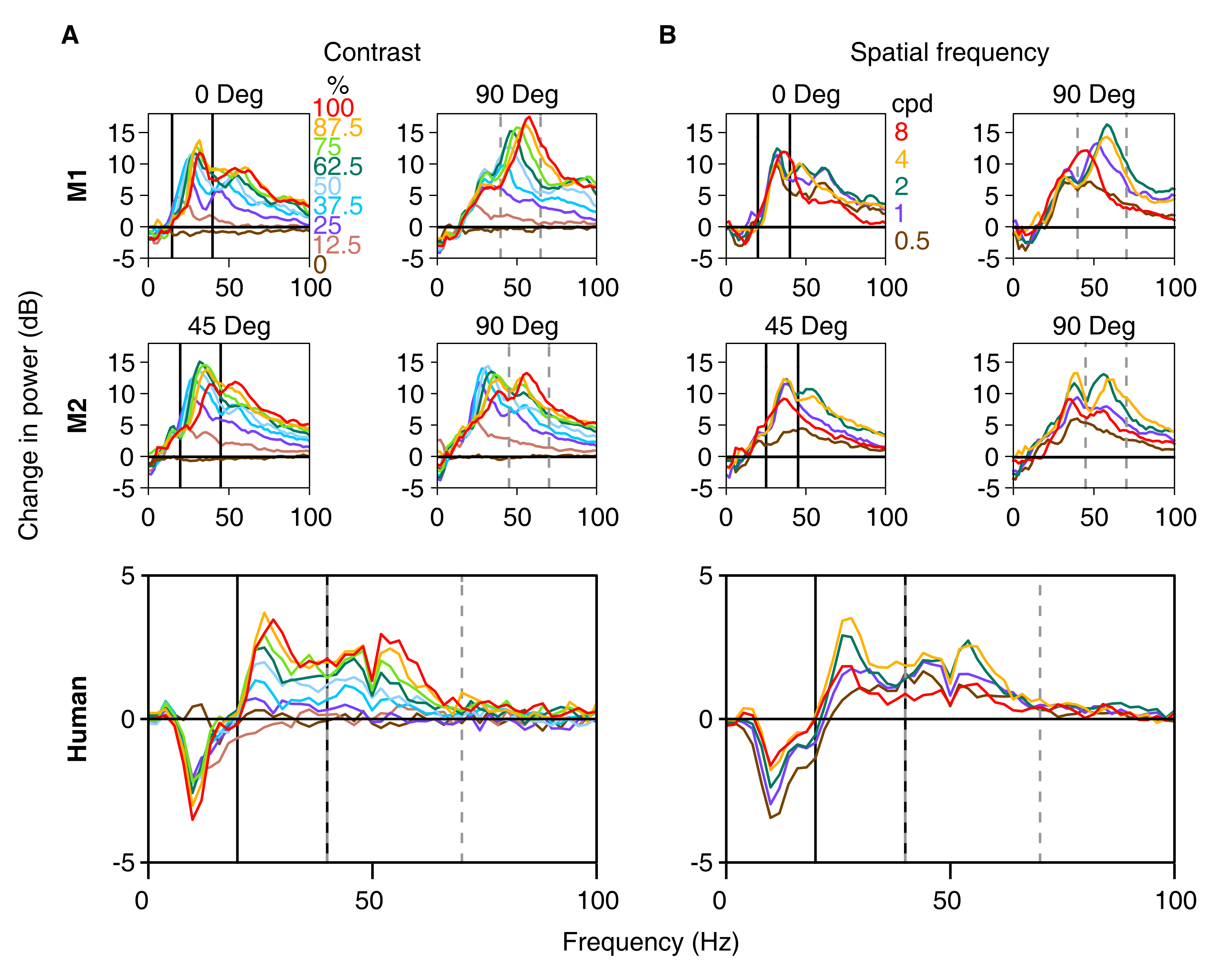

Supplement: Figure 3-1 [file zns999180605so5.tif]

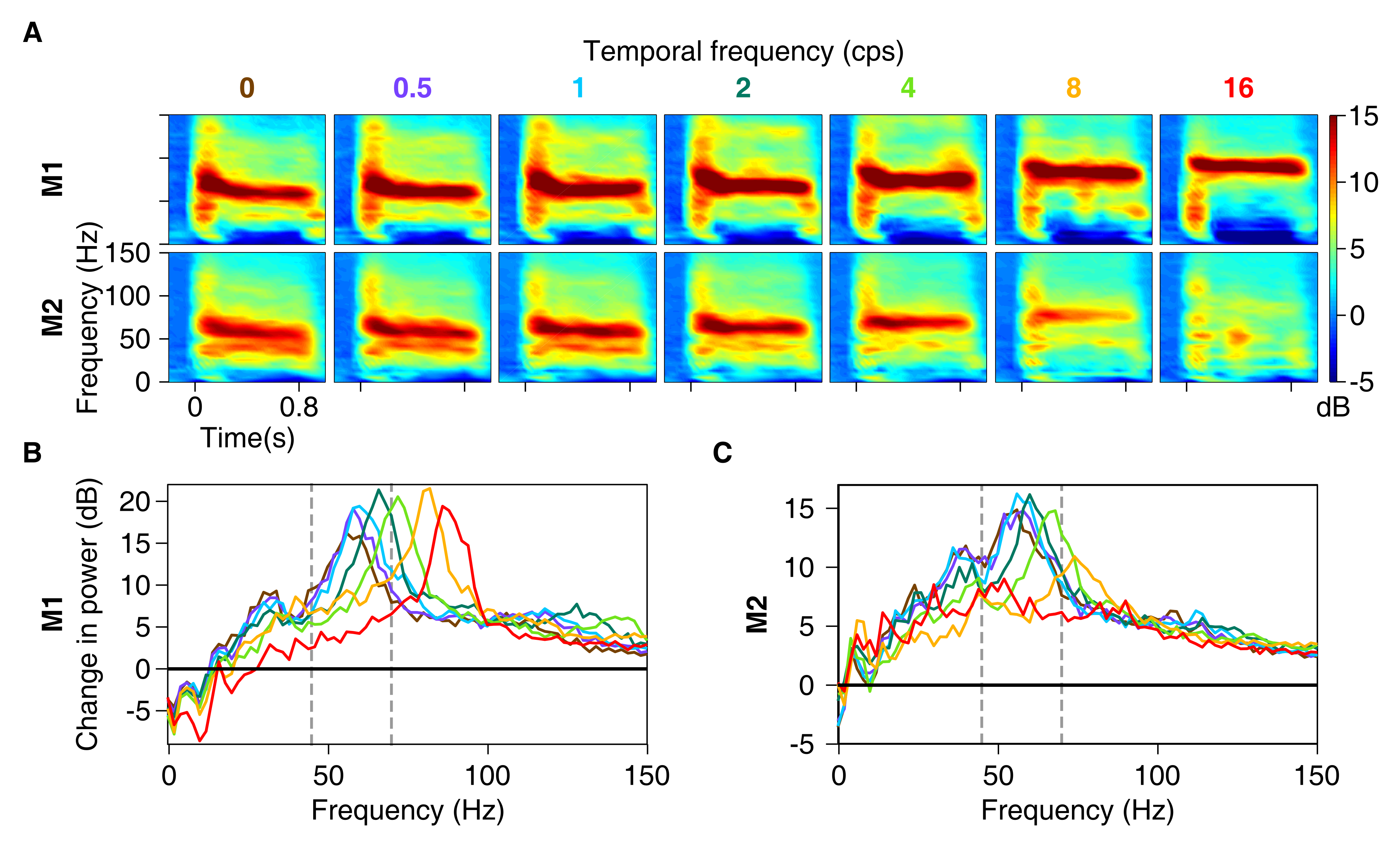

Supplement: Figure 4-1 [file zns999180605so6.tif]

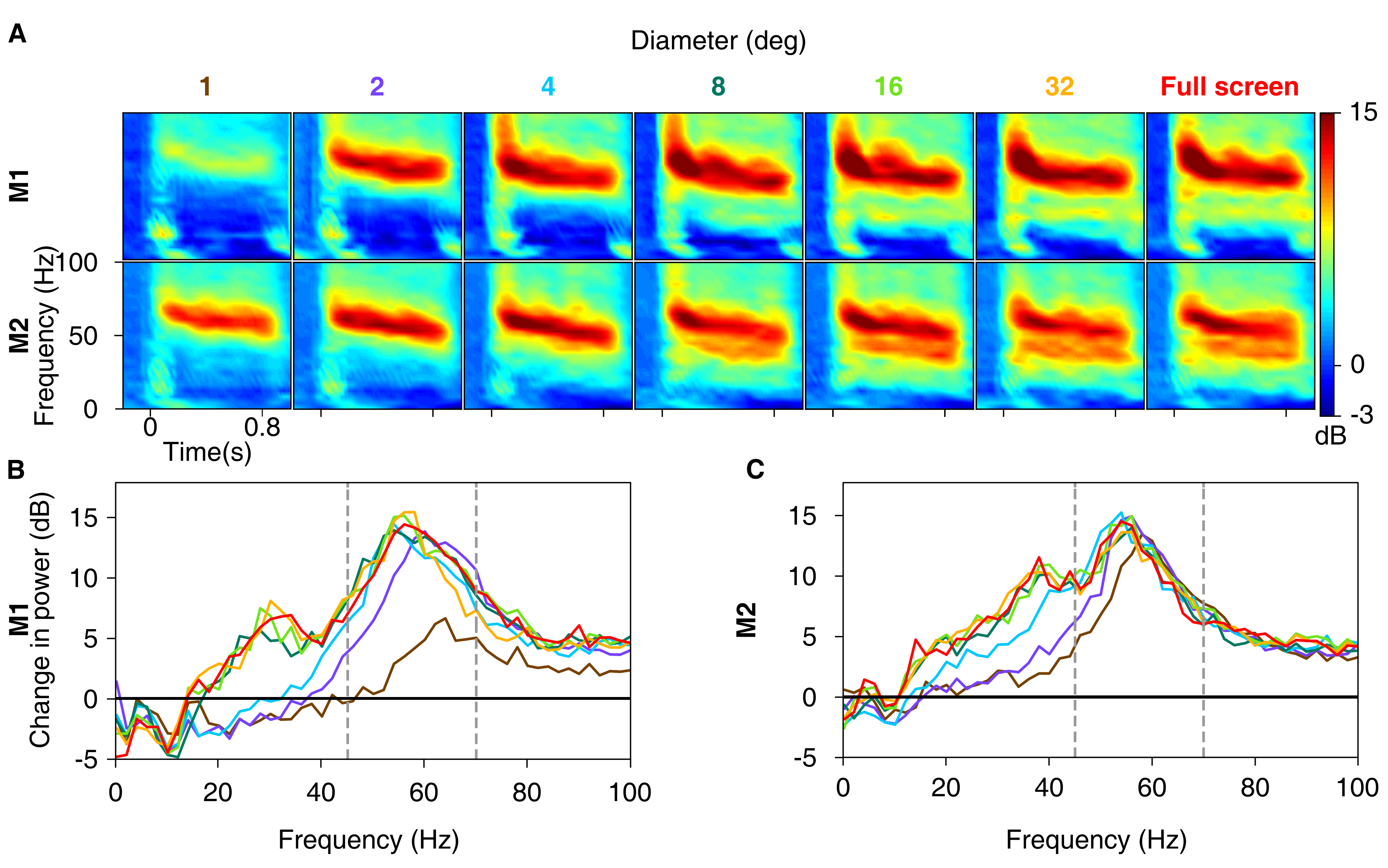

Supplement: Figure 5-1 [file zns999180605so7.tif]
